# Foods may modify responsiveness to cancer immune checkpoint blockers by altering both the gut microbiota and activation of estrogen receptors in immune cells

**DOI:** 10.3389/frmbi.2022.1049688

**Published:** 2022-12-12

**Authors:** Leena Hilakivi-Clarke, Vivek Verma, Maddie McDermott, Pal Koak, Fabia de Oliveira Andrade

**Affiliations:** The Hormel Institute, University of Minnesota, Austin, MN, United States

**Keywords:** cancer, immune checkpoint inhibitors, gut microbiota, diet, estrogen receptors

## Abstract

Estrogen receptor alpha positive (ERα+) breast cancers are refractory to immune checkpoint blocker (ICB) monotherapy, while ICBs are part of a standard of care for triple negative breast cancers (TNBCs). Besides tumor ERα expression, another difference between the two types of breast cancers is that only ERα+ patients exhibit elevated tumor estradiol (E2) levels, compared with surrounding normal tissue. Recent evidence suggests that inhibition of ERα or activation of ERβ or G protein-coupled estrogen receptor (GPER) in immune cells in the tumor microenvironment (TME) increases tumor CD8+ T cell infiltration and boosts cancer ICB response. Ovarian and adipose-produced estrogens activate all three ERs equally, but plant estrogens (phytochemicals) preferentially activate ERβ or GPER. The gut microbiota is a key player in determining response to ICBs, and high abundance of Firmicutes and high fecal levels of short chain fatty acids (SCFAs) that are mainly produced by Firmicutes, are linked to improved effectiveness of ICB therapy. Interestingly, the gut microbiota of ERα+ breast cancer patients contain significantly lower abundance of Firmicutes species than the gut microbiota of TNBC patients. Many factors modify the gut microbiota, especially diet. The gut microbiota altering diets include (i) foods high in ERβ and GPER activating plant phytochemicals or (ii) SCFAs producing fiber that also reduces circulating estrogen levels, (iii) estrogen levels reducing fasting/caloric restriction, or (iv) ketogenic diet which reduces fecal SCFA levels but increases hepatic production of SCFA receptor activating ketone bodies. It is thus possible that certain foods or dietary patterns can modify both the gut microbiota and activation of the estrogen receptors in the tumor immune cells, and consequently regulate the effectiveness of ICB therapy against cancers.

In optimal circumstances, activated CD8+ T cells infiltrate the tumor microenvironment (TME) and kill cancer cells. However, before CD8+ T cells can complete eliminating cancer cells, they often become exhausted, partly by activation of immune checkpoints. Immune checkpoints, including programmed cell death 1 (PD1), cytotoxic T lymphocyte-associated protein 4 (CTLA-4), lymphocyte-activation gene 3 (LAG-3), and T cell immunoglobulin and mucin-domain containing-3 (TIM-3), are expressed in immune cells. Their task is to prevent immune activation targeted toward self-antigens (Sharma and Allison, 2015); i.e., prevent autoimmune diseases. Blocking the immune checkpoints by various antibodies is the most used form of cancer immunotherapy, and is highly effective in a subgroup of cancer patients (Dong et al., 2017). Cancers that are responsive to immune checkpoint blocker (ICB) therapy include advanced melanoma, non-small cell lung cancer (NSCLC), cutaneous squamous cell carcinoma, urothelial cancer, renal cell cancer (RCC), refractory Hodgkin lymphoma, hepatocellular carcinoma (HCC), gastric cancer, and triple-negative breast cancer (TNBC). Accordingly, ICBs have been integrated into the standard of care regimens for these cancer types. However, only approximately 20-30% of all ICB-treated patients with these cancers respond to ICBs given as a monotherapy (Lu et al., 2015). Further, patients who initially respond can become resistant to ICBs.

The factors contributing to primary and secondary resistance to ICBs are multifaceted and include both tumor intrinsic factors and factors arising from a complex interplay between cancer and its microenvironment (Samstein et al., 2019). Among the factors are low T-cell infiltration levels, low mutational load, T-cell receptor clonality and gene expression (Snyder et al., 2014; Van Allen et al., 2015; Gibney et al., 2016; Sharma et al., 2017; Jenkins et al., 2018). Correct scheduling of ICBs also is critical (Verma et al., 2019). Consequently, the approaches which have been attempted to reverse ICB resistance involve either a modification of the tumor or altering the TME to enhance its immunogenicity (Murciano-Goroff et al., 2020).

The gut microbiota which is mostly composed of bacteria and their viruses (phages), archaea and microeukaryotes and has a mutually beneficial bi-directional relationship with the host (e.g., human), is the main regulator of immune responses (Cani, 2018). One of the first indications that the gut microbiota is involved in impacting tumor responsiveness to ICBs came from an observation that genetically similar C57BL/6 mice which received B16.SIY melanoma cell allografts responded better to PDL1 monoclonal antibody (mAb) therapy if the mice were obtained from Jackson Laboratory (JAX) than from Taconic Farms (TAC) (Sivan et al., 2015). Mice from the two different vendors are known to exhibit significant differences in their commensal microbes (Ivanov et al., 2009). JAX and TAC mice also differed regarding tumor specific T cell responses: the better-responding JAX mice had higher intra-tumoral CD8^+^ T cell accumulation. The difference in ICB response and tumor immune response was eliminated by co-housing mice or performing fecal microbiota transfer (FMT) between JAX and TAC mice. Reduced abundance of genus *Bifidobacterium* was identified as a potential key player for impaired ICB responsiveness in TAC mice: supplementation of TAC mice with a cocktail of multiple *Bifidobacterium* species improved their response to ICB therapy (Sivan et al., 2015). Three key human studies were published in 2018 confirming the findings obtained in mice showing that the composition of the gut microbiota was causally linked to the ICB responsiveness (Routy et al., 2018; Gopalakrishnan et al., 2018; Matson et al., 2018).

Another main regulator of tumor immune response is estrogens and their receptors in the TME (Rothenberger et al., 2018; Schafer et al., 2022). Only a handful of preclinical studies have investigated whether estrogens modify the ICB response (Marquez-Garban et al., 2019; Chakraborty et al., 2021). Breast cancers which do not express the estrogen receptor alpha (ERα) are responsive to ICBs, while breast cancers expressing ERα are not responsive (Rugo et al., 2018; Planes-Laine et al., 2019; Goldberg et al., 2021). This could suggest the TME is different in ERα+ and TNBCs, and the difference impairs ICB responsiveness in ERα+ breast cancer. To support this, ERα+ breast cancers exhibit lower T cell infiltration than TNBCs, including lower numbers of anti-tumor CD8+ T cells (Stanton et al., 2016). One factor which could dampen T infiltration in ERα+ breast cancers is the higher estrogenic environment in these cancers, compared with TNBC (Pasqualini et al., 1996). The concentration of the most potent estrogen – 17β-estradiol (E2) – is higher in ERα+ breast tumors than in normal breast tissue (reviewed in Yaghjyan and Colditz (Yaghjyan and Colditz, 2011). TNBCs do not contain more E2 than normal breast tissue (van Landeghem et al., 1985; Lonning et al., 2009). Local factors, such as tumor adipogenesis, might explain the higher tumor E2 levels in ERα+ breast cancer. ERα+ breast cancers exhibit significantly higher adipogenesis than TNBCs (Oshi et al., 2021).

Since circulating E2 levels correlate with breast tissue E2 levels (Depypere et al., 2015), factors that increase E2 concentrations might impair ICB responsiveness. Diet can modify circulating estrogen levels: either to reduce them (caloric restriction) or increase them (dietary factors which activate aromatase). Diet might also affect estrogenicity by affecting the gut microbiota through increasing β-glucuronidase (GUS) production (Baker et al., 2017). GUS is an enzyme that deconjugates estrogens into their active forms, thus leading to an increase in circulating E2 levels.

We will focus here on two diet-related mechanisms which potentially alter ICB responsiveness: the gut microbiota and estrogenicity in the TME. A potential relationship between diet and gut microbiota has been reviewed extensively (Maslowski and Mackay, 2011; David et al., 2014b; Singh et al., 2017; Suez et al., 2018; Zmora et al., 2018; Burr et al., 2020). In contrast, relatively little is known about diet and ICB response (Szczyrek et al., 2021; Spencer et al., 2021). Similarly, the possibility that estrogenic foods modify cancer patients’ response to ICBs has not received much attention. Estrogenic foods include those that increase circulating estrogen levels or contain compounds that bind and activate estrogen receptors. There also might be a link between estrogenic foods and gut microbiota. In this review, we will discuss the role of diet, especially estrogenic diet, in affecting the gut microbiota, immune responses in the TME, and the interaction between gut microbiota and estrogenic TME in affecting the response to ICB therapies.

## Gut microbiota as a modifier of ICB responsiveness

### The gut microbiota

The human body is composed of an equal number of human and microbial cells (Sender et al., 2016), and most of the microbiota reside in the gut. However, the number of genes from all the microorganisms in the human body is at least 10 times higher than the genes present in human cells (Sender et al., 2016). Microbiome is defined as microbiota, its metabolites, genes and other components (Berg et al., 2020). In contrast to the human genome which is 99.9% identical across all humans (Wheeler et al., 2008), the composition of the gut microbiota is only 10-20% similar among individuals (Turnbaugh et al., 2010). Thus, each person has a unique gut microbiota (Rinninella et al., 2019). The gut microbiota is established early in life during the first three years of life and thereafter remains stable (Wu et al., 2011). Although many factors in adult life can influence it, such as diet and physical activity, their impact on the gut microbiota tends to last only for the period an individual is exposed to them (David et al., 2014a; Allen et al., 2018; Leeming et al., 2019). For example, in studies investigating the impact of dietary intervention on the gut microbiota, diet-induced changes in the gut microbiota composition are generally lost during a 4-week washout-period between experimental and control diet (Leeming et al., 2019). However, it is not known if long-term dietary change, such as switching from a carnivore to a vegetarian, will permanently alter the microbial taxa (Leeming et al., 2019).

The gut microbiota in humans is dominated by the phyla Firmicutes and Bacteroidetes (these two compose 90% of the gut microbiota (Almeida et al., 2019), Actinobacteria, Proteobacteria and Verrucomicrobia (Lagkouvardos et al., 2016; Almeida et al., 2019). Some of these phyla were recently renamed, but the new names remain somewhat contradictory and to avoid confusion, we use the old names here. Each phylum is subdivided into class, order, family, genus and species. The Firmicutes phylum is composed of over 200 genera, including *Clostrodium* (most abundant), *Lactobacillus*, *Enterococcus*, and *Ruminococcus*. *Bacteroides* and *Prevotella* are the main genera of Bacteroidetes phylum. The Actinobacteria phylum is much less abundant than Firmicutes and Bacteroidetes, and *Bifidobacterium* genus is its main member. Members of Proteobacteria phylum include many pathogens, such as *Escherichia*, *Salmonella*, *Helicobacter* and *Vibrio.* Proteobacteria tend to have unique rather than overlapping functions, and the variability in Proteobacteria gene expression may signal for gut dysbiosis (Bradley and Pollard, 2017). Verrucomicrobia represents less than 1% of the gut phyla, and *Akkermansia muciniphila* (*A. muciniphila*) used to be the sole species of this phylum until recently.

The gut microbes provide several critical functions for the host (Derrien et al., 2017): they impact nutrient metabolism, especially carbohydrate metabolism, produce neurotransmitter precursors, such as for dopamine, norepinephrine and GABA or produce metabolites that promote the synthesis and release of serotonin by enteroendocrine cells, maintain the structural integrity of the gut mucosal barrier, protect against pathogens, and are key immunomodulators (Cani, 2018). Gut microbiota also modifies mitochondrial functions in host cells *via* microbial metabolites (Schonfeld and Wojtczak, 2016; Jackson and Theiss, 2020). When assessing the gut microbial composition and its relationship with health, two markers have emerged. Healthy individual’s gut microbiota (i) exhibits high alpha-diversity, reflecting a presence of high number of different bacterial genera and species (Reese and Dunn, 2018; Rinninella et al., 2019), and (ii) produces high levels of short chain fatty acids (SCFAs) (Morrison and Preston, 2016; Schirmer et al., 2018; Li et al., 2018). A third marker might be Firmicutes to Bacteroidetes (F/B) ratio (Molist et al., 2012), although the direction of the ratio and health remains controversial. In connection with obesity that increases the ratio (Turnbaugh et al., 2006; Turnbaugh et al., 2009), high F/B ratio is a potential marker of gut dysbiosis. However, low F/B ratio is linked to inflammatory gut diseases (Morgan et al., 2012) and consumption of plant-based foods considered as health-promoting increases the ratio (Lin et al., 2013; Szczyrek et al., 2021). For these reasons, F/B ratio is not appreciated by many in the field of the gut microbiota and health.

### Observations supporting a causative link between the gut microbiota and ICB response

#### The gut microbiota and the response to ICBs: Human studies

Three years after the discovery suggesting that the gut microbiota affects ICB response in mice (Sivan et al., 2015), three human studies were published showing that the gut microbiota influences the response to ICBs (Routy et al., 2018; Gopalakrishnan et al., 2018; Matson et al., 2018). In the study conducted by Routy et al., NSCLC, RCC or urothelial patients treated with antibiotics before, at the time, or after receiving ICB had significantly shorter progression-free survival and overall survival than patients who did not need antibiotics (Routy et al., 2018). Antibiotics are known to inhibit the abundance of commensal gut microbes (Ramirez et al., 2020). Patients not treated with antibiotics had higher gut levels of Firmicutes phylum and two bacterial genera: *Akkermansia* and *Alistipes*. Germ-free (GF) mice that received FMT from ICB responding or non-responding patients themselves responded better to anti-PD1 mAb therapy if the FMT originated from responding patients. Further, *A. muciniphila* improved response to anti-PD1 mAb therapy in mice receiving FMT from non-responding patients. In this study, the improved response was linked to increased tumor ratio of CD4+ derived T helper 1 (Th1) cells to Foxp3/T regulatory (Treg) cells (Routy et al., 2018).

Data from the other two human studies (Gopalakrishnan et al., 2018; Matson et al., 2018) also indicated a causative link between the gut microbiota and response to ICBs. However, the changes in the gut microbiota were not similar in the two studies or to those of the Routy study (Routy et al., 2018). This is not surprising, since multiple bacterial species have overlapping functions, and consequently, different sets of bacteria could similarly alter tumor immune infiltration and immune functions to improve response to ICB therapy. In the Gopalakrishnan study (Gopalakrishnan et al., 2018), the *Faecalibacterium* genus from the *Ruminococcaceae* family of Firmicutes was present at a significantly higher abundance in the responders than non-responders. *Bacteroidales* order and the genera *Prevotella* under phylum Bacteroidetes was over-represented in the non-responders. In the Matson study (Matson et al., 2018), *B. longum*, *Collinsella aerofaciens* (both belong to phylum Actinobacteria), and *Enterococcus faecium* (Firmicutes) species were enriched among responders. Importantly, the gut microbiota that favored response to ICBs in these two studies was associated with increased antigen processing and presentation, higher infiltration of CD8+ T cells and lower infiltration of Foxp3/T cells in the TME.

Additional seven human studies and the three studies outlined above were included in a comprehensive review by Oh et al. (Oh et al., 2021) to assess a connection between the human gut microbiota and responsiveness to immunotherapies. Patients in the studies had melanoma, HCC, NSCLC and RCC. In six of the studies, high alpha-diversity was linked to clinical response to ICBs, and in three studies, high abundance of Firmicutes predicted ICB responsiveness. High abundance of *Lachnospiraceae* and *Ruminococcaceae* families of Firmicutes phyla predicted high responsiveness in three and four studies, respectively. These two families are the main SCFA producers (Louis et al., 2014; P et al., 2016; Louis and Flint, 2017). At the genus level, high abundance of *Fecalibacterium* from *Ruminoccaceae* family was seen in four studies linked to ICB response, and in three studies high abundance of *Bacteroides* was linked to ICB refractoriness. Finally, of various bacterial species, the abundance of *Fecalibacterium prausnitzii* was upregulated in ICB responsive cancer patients in four studies, *Bifidobacterium longum* in three studies, and *A. muciniphila* was upregulated in ICB responsive HCC, NSCLC and RCC patients. Both *A. muciniphila* and *Bifidobacterium longum* are SCFA producing bacterial species (Derrien et al., 2004; Riviere et al., 2016; Louis and Flint, 2017). Another systematic review (Huang et al., 2021) concluded that patients with solid tumors who had elevated abundance of Firmicutes and Verrucomicrobia phyla responded best to ICBs.

**Figure 1:**
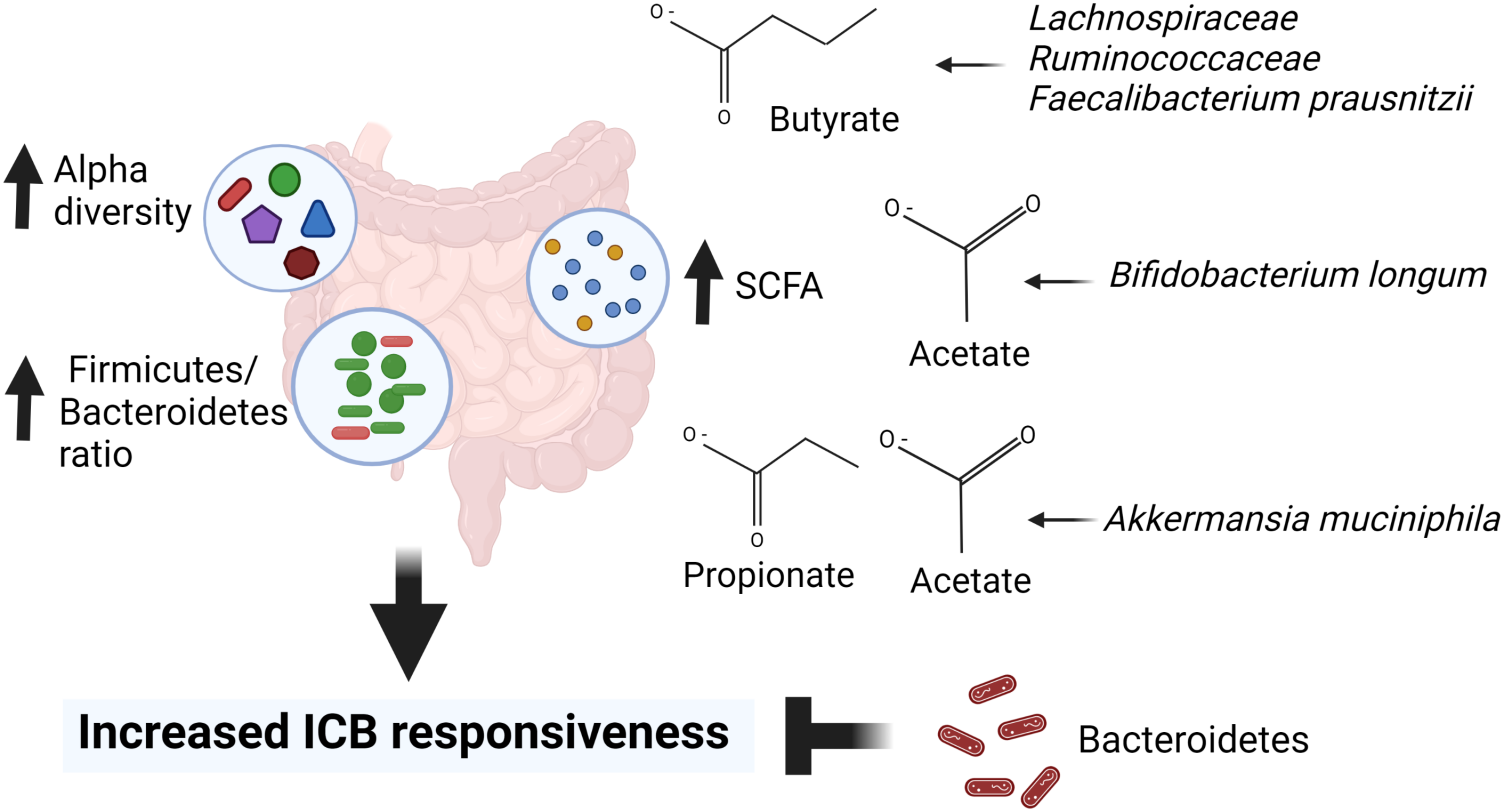
Gut microbiota markers for immune checkpoint blocker (ICB) responsiveness. High alpha diversity, high Firmicutes to Bacteroidetes ratio (F/B) and high abundance of short chain fatty acids (SCFAs) are linked to responsiveness to ICB therapy. In addition, ICB responders have higher abundance of SCFA producing bacteria including *Lachnospiracea, Ruminococcaceae, Faecalibacterium prausnitzi, Bifidobacterium longum* and *Akkermansia muciniphila* than ICB refractory cancer patients. Created in Biorender.com.

Most recently Lee et al. (Lee et al., 2022) sequenced stool samples obtained from five different melanoma patient cohorts before they were treated with ICBs. Two of the cohorts contained over 50 patients each but the remaining three were considerably smaller. A total number of patients in the five studies was 165. The larger two cohorts were called PRIMM cohorts, and one composed of patients treated in the United Kingdom (UK) and the other of patients treated in the Netherlands (NL). The quantitative taxonomic gut microbiome composition, as defined by the response definition in three previous microbiome–ICB studies (Frankel et al., 2017; Routy et al., 2018; Wind et al., 2020) was different between the UK patients (p=0.05), but not in the NL patients (p=0.61). However, alpha-diversity was higher in the responding NL patients but not in the UK patients. When using the prediction ability of the combination of taxonomic and functional features of the microbiome, both PRIMM studies were able to segregate responders and nonresponders, albeit the predictive signatures in the studies were different from each other. Thus, these findings support the earlier studies indicating that there are no global bacterial signatures predictive of ICB responsivess. When the investigators searched for bacterial species in their cohorts and available databases that were related to response to ICB therapy in multiple studies, they identified bacteria that produce SCFAs (Lee et al., 2022). **Figure 1** summarizes the observed links between the gut microbiota and ICB responsiveness from the studies outlined above. Although it is apparent that there is no universal gut microbiota composition that is predictive of ICB responsiveness, across the studies responders have higher abundance of SCFA producing bacteria that mostly belong to Firmicutes phyla, *Bifidobacterium* genus or *A. muciniphila* species.

#### Fecal microbiota transfer

The most direct evidence linking the gut microbiota to ICB response comes from the FMT studies. In addition to the studies in which FMT is prepared from ICB responding or refractory patients, and it is gavaged to mice which have previously undergone their own gut microbiota depletion (Routy et al., 2018; Gopalakrishnan et al., 2018; Matson et al., 2018), two recent studies evaluated the feasibility of treating PD1-refractory melanoma patients with the FMT obtained from responsive patients (Baruch et al., 2021; Davar et al., 2021). Both studies established the safety and feasibility of the approach. Though one of the studies was limited by a small number of patients studied (two donors and 10 recipients were included) (Baruch et al., 2021), 3 of 5 FMT recipients from a single donor became responsive to ICB. FMT from the second donor failed to improve responsiveness in the remaining 5 non-responding patients.

The study by Davar (Davar et al., 2021) included 15 PD1 treatment refractory patients and multiple donors. Of the 15 patients, six exhibited a clinical response upon re-treatment with anti-PD1 after receiving responder derived FMT. The gut microbiota of these six FMT responding patients contained a significantly enriched taxa of the phylum Firmicutes, especially SCFA-producing *Lachnospiraceae* and *Ruminococcaceae* families, and phylum Actinobacteria (*Bifidobacteriaceae* and *Coriobacteriaceae* families). *Bifidobacteria* also is SCFA producer (Riviere et al., 2016). The bacteria that were decreased in the responders belonged mostly to phylum Bacteroidetes. Moreover, FMT responding patients had higher levels of activated CD8+ T cells with higher cytolytic functions than FMT non-responders in the circulating blood cells and tumor biopsies (Davar et al., 2021). This is consistent with activation of CD8+ T cells seen in anti-PD1 mAb responding cancer patients (Daud et al., 2016; Gide et al., 2019). Furthermore, the proportion of immunosuppressive myeloid cells was higher in the patients who remained ICB refractory. Finally, patients who responded to ICB retreatment after receiving FMT exhibited changes in circulating cytokines and chemokines, such as lower levels of CXCL8 (IL8) (Davar et al., 2021) which is needed for immunosuppressive myeloid derived suppressor cell (MDSC) activation (Tobin et al., 2019). These studies provide convincing evidence that the composition of the gut microbiota is involved in determining response or resistance to ICBs, potentially through mechanisms which alter the tumor immune microenvironment.

## Probiotics and prebiotics as modifiers of the ICB response

### Probiotics

There is considerable interest in the potential health benefits of pro- and prebiotics (Liu et al., 2015b; Dey et al., 2019). Probiotics are live bacteria that are present for example in fermented foods, such as yogurt, sauerkraut and kimchi. These food products are traditionally consumed in many European and Asian countries. However, in the U.S. probiotics often mean supplements taken as pills, such as those containing various strains of *Bifidobacterium* and *Lactobacillus*. It is not clear how effective and safe probiotic supplements are in altering the gut microbiota (Xavier et al., 2020). Although these supplements effectively colonize the gut of GF mice, microbiota in conventionally housed mice exhibit a marked resistance to probiotics (Zmora et al., 2018). In humans, response to probiotics is highly individualized (Zmora et al., 2018), but so is also the response to diet (Kolodziejczyk et al., 2019). Further, when the gut microbiota composition is disturbed, for example by antibiotics or stress, probiotics might prevent individual’s healthy gut microbiota from being re-established (Suez et al., 2018). For this reason, probiotic supplements may not be an ideal way to improve the composition of the gut microbiome, especially to prevent cancer or in cancer patients. It is also concerning that commercial probiotics have been linked to bacteremia (Doron and Snydman, 2015; Lu et al., 2021), and that some probiotics have been shown to initiate cancer in an organoid culture (Pleguezuelos-Manzano et al., 2020).

Several animal studies have investigated the ability of probiotics to impact ICB responsiveness. When poorly ICB responding C57BL/6 mice from Taconic were supplemented with a cocktail of multiple *Bifidobacterium* species, their responsiveness was improved (Sivan et al., 2015). Similarly, anti-PD1 non-responsive mice allografted MCA-205 sarcoma cells became responsive when treated with *A. muciniphila* (Routy et al., 2018), and mice with orthotopically injected RENCA cells started responding to anti-PD1 and anti-CTLA4 when they were treated with *A. muciniphila* and *Bacteroides salyersiae* (Derosa et al., 2020). In humans, Japanese NSCLC patients who were co-treated with ICBs and *Clostridium butyricum* MIYAIRI 588 strain that is commonly used in Japan to reduce symptoms related to antibiotic-induced dysbiosis improved the ICB responsiveness, both in antibiotic users and those not taking antibiotics (Tomita et al., 2020). In an on-going clinical trial involving Chinese liver cancer patients, the effectiveness of a probiotic *Lactobacillius rhamnous* to promote anti-PD1 responsiveness is currently being tested (NCT05032014). *Lactobacillius rhamnous* was chosen because it inhibits colon cancer growth and activates colonic CD8+ T cells in mice (Owens et al., 2021), and potentiates the effectiveness of anti-PD1 against allografted colon cancer growth in syngeneic Balb/c mice (Gao et al., 2021). However, since the metabolite of *Lactobacillius rhamnous* is lactate/lactic acid, and in a recent study lactic acid was found to specifically upregulate PD1 in Treg cells but not in CD8+ T cells as well as cause a failure of anti-PD1 therapy against liver cancer (Kumagai et al., 2022), there is a concern of the outcome of the study.

Despite the positive results from mouse studies and one human clinical trial outlined above, probiotic supplements are not advised to be used to reverse the resistance to ICBs (Lee et al., 2021a). The importance of caution is highlighted by the results obtained in an observational study assessing possible connections between diet, probiotics and ICB response among melanoma patients (Spencer et al., 2021). Melanoma patients who reported having taken probiotic supplements during the ICB therapy had significantly shorter survival than other melanoma patients, even when they also consumed high levels of fiber in their diet which without probiotics was linked to significantly improved survival (Spencer et al., 2021). Whether probiotics naturally present in foods, such as in fermented foods, would improve ICB responsiveness should be investigated. For example, foods containing *Lactobacillius rhamnous* include kefir, kimchi, sauerkraut, sourdough bread, and yogurt. These foods are likely to be safer when given to cancer patients treated with ICBs than *Lactobacillius rhamnous* supplement.

### Prebiotics

Many definitions of prebiotics exist (Davani-Davari et al., 2019). The most commonly accepted is that a prebiotic is “a selectively fermented ingredient that results in specific changes in the composition and/or activity of the gastrointestinal microbiota, thus conferring benefit(s) upon host health” (Gibson et al., 2010). Solubility and fermentability are not a defining criterion for a prebiotic compound, but rather prebiotics are those food ingredients that (i) avoid being absorbed in the gastrointestinal tract, (ii) can be fermented by intestinal microbiota, and (iii) selectively stimulate the intestinal bacteria (Gibson et al., 2010). Simply put, prebiotics are dietary fibers, but not all dietary fibers are prebiotics (Slavin, 2013). Dietary fibers have recently been redefined as microbiota-accessible carbohydrates (MACs).

A recent study compared the effectiveness of two soluble dietary fibers – inulin and mucin – against colon cancer and melanoma in C57BL/6 mice (Li et al., 2020). Inulin is a naturally occurring fructosyl polymer with chain-terminating glucosyl residues. Its dietary sources include garlic, onion, leek, rye, barley, banana and many root vegetables. Mucins are highly decorated with polysaccharides composed of various core structures similar to those found in Lewis blood type antigens, including various sugars. Mucins are present for example in human and bovine milk, and thus in many dairy products. Inulin and mucin both inhibited melanoma cell growth in syngeneic mice, and when given in combination, they were more effective than either prebiotic alone (Li et al., 2020). Only inulin inhibited colon cancer. Both prebiotics affected tumor immune responses. Inulin activated CD4+ and CD8+ T cells, while mucin stimulated antigen presentation. The two prebiotics had both similar and different effects on the gut microbiota. As expected, among the bacterial phylotypes increased by inulin and mucin were taxa that produce SCFA butyrate. Not all findings support cancer therapeutic effects of prebiotics: in a study using multiple strains of dysbiotic mice, soluble inulin induced icteric hepatocellular carcinoma (HCC) (Singh et al., 2018). Thus, inulin supplementation might protect against some types of cancers and promote some others, suggesting caution with taking prebiotics as supplements. These two studies did not investigate if inulin or mucin improved response to ICBs.

Very few studies have investigated if prebiotics impact the effectiveness of ICBs. It was recently found that in several murine cancer models, inulin potentiated the ability of anti-PD1 mAb to inhibit tumor growth (Han et al., 2021). As already mentioned, in a clinical study involving melanoma patients treated with ICBs, patients who consumed the highest levels of dietary fiber exhibited the best response to ICBs (Spencer et al., 2021). This finding suggests that prebiotics in foods might be an important tool to prevent ICB refractoriness. However, dietary fiber supplementation alone may not be sufficient. MACs are low in Western diet, and when Western diet is consumed over multiple generations, irreversible loss of bacterial diversity takes place, including a reduction in SCFA producing bacteria (De Filippo et al., 2010). Animal study showed that this multigenerational loss cannot be recovered solely by consumption of MACs, but missing bacterial taxa also needs to be replaced (Sonnenburg et al., 2016). This may also be true for humans, although the finding that dietary fiber intake could not reverse the adverse effect of probiotics on ICB responsiveness in an observational human study (Spencer et al., 2021) suggest that in the context of ICB therapy probiotics should not be consumed.

## Potential mechanisms through which the gut microbiome might affect ICB response: Microbial metabolites and the immune response

In the studies described above, patients who responded to ICBs had a gut bacterial composition that was linked to optimal activation of CD8+ T cells. Gut lamina propria houses different arms of the adaptive immune system consisting of naïve CD4+ T cells that can differentiate to immunosuppressive Foxp3/Tregs, Th2, and Th17 immune cells, or anti-tumor Th1 cells. The importance of the gut microbiota for normal immunity is highlighted in mice which are housed in GF environment; they develop several immune deficits, including loss of Tregs which are critical to control autoimmunity (Round and Mazmanian, 2009; Gianchecchi and Fierabracci, 2018; Kitz et al., 2018). A recent study identified an 11 bacterial strain signature, isolated from a fecal sample of one healthy human donor, that induced CD8+ T cell activation in the colonic lamina propria and systemically in GF mice (Tanoue et al., 2019). Importantly, these 11 bacterial strains seemed to be working together to activate CD8+ T cells, because depleting even one of the strains reduced CD8+ T cell activation. Fecal samples obtained from five other donors did not initiate CD8+ T cell immune response in GF mice. Whether these 11 bacteria that are all rare members of the gut microbial community (Tanoue et al., 2019) and activated CD8+ T cells in GF mice also can improve responsiveness to ICB is not known.

### Short chain fatty acids

The gut microbiome derived metabolites may explain how the gut microbiota can modify immune cell activation and responsiveness to various ICB therapies (Helmink et al., 2019; Mager et al., 2020; Griffin et al., 2021). Of these bacterial metabolites, SCFAs (acetate, butyrate and propionate) have received most attention. SCFAs have a chain length of up to six carbon atoms. They are produced by the gut microbiota in the large intestine as a result of the anaerobic fermentation of polysaccharides present in carbohydrates, such as in dietary fiber (Miller and Wolin, 1996; Louis and Flint, 2009). The gut bacteria which generate most of the butyrate are Firmicutes, in particular families *Ruminococcaceae* and *Lachnospiraceae* (Louis et al., 2014; Louis and Flint, 2017). High *Ruminococcaceae* and *Lachnospiraceae* abundance are both associated with ICB responsiveness in human studies (Oh et al., 2021). *A. muciniphila* produces both propionate and acetate (Derrien et al., 2004; Louis and Flint, 2017) and *Bifidobacteria* produces acetate (Riviere et al., 2016). *Prevotella* of the Bacteroidetes phylum was found to produce acetate (Koh et al., 2016). However, other studies have reported that several *Prevotella* species inhibit the production of acetate, butyrate and propionate (Lucas et al., 2018), including a novel *Prevotella intestinalis* strain (Iljazovic et al., 2021).

SCFAs have many biological functions. In addition to regulating the immune system and inflammatory response (Morrison and Preston, 2016; Sun et al., 2017; Schirmer et al., 2018), SCFAs are the main energy source for the gut microbiota (Rios-Covian et al., 2016), they maintain gut membrane integrity (Correa-Oliveira et al., 2016), improve mitochondrial metabolism (Schonfeld and Wojtczak, 2016; Jackson and Theiss, 2020), also in CD8+ T cells (Bachem et al., 2019), and are histone deacetylase (HDAC) inhibitors (mainly butyrate). Thus, as HDAC inhibitors SCFAs can activate epigenetically silenced immune genes (Licciardi et al., 2011; Yuille et al., 2018; Li et al., 2018). SCFAs affect target cells both through their receptors, and independent of the receptors, such as through various monocarboxylate transporters, including Slc26a1 and Slc5a8. SCFAs bind to G protein-coupled receptors (GPRs): free fatty acid receptor 2 (FFAR2, also called GPR43), FFAR3 (or GPR41), hydroxycarboxylic acid receptor 2 (HCA2 or GPR109A) and olfactory receptor 78 (OLF78). These receptors are expressed in the colonic epithelium, and gut immune cells and enteroendocrine cells (Schlatterer et al., 2021). In addition, they are expressed in many cells and tissues outside the gut microenvironment, including immune cells, endocrine tissues (van der Hee and Wells, 2021b) and possibly even in the brain (Silva et al., 2020). In principle, SCFAs can reach their receptors *via* portal vein to the liver and then to circulation. However, most of propionate and butyrate are cleared in the liver, but acetate can reach detectable levels in the venous human serum (Bloemen et al., 2009). How much of SCFAs produced by the gut microbiota remain detectable in the periphery and CNS remains unclear. SCFAs can cross the blood-brain barrier, and in mice supplementation with butyrate producing *Clostridium butyricum* increased brain butyrate levels an order of magnitude more than in the peripheral blood (Liu et al., 2015a). Of the SCFA receptors, GPR43 and GPR109A, and less so GPR41, are expressed in immune cells and have anti-inflammatory properties (Deleu et al., 2021). These receptors are also expressed in the adipose tissue and epithelial cells of various tissues, and they regulate glucose metabolism, energy expenditure and mitochondrial function (van der Hee and Wells, 2021a; Correa-Oliveira et al., 2016; Kim, 2021). GPR41 is expressed in the sympathetic ganglia where it regulates appetite and heart rate (Correa-Oliveira et al., 2016). OLF78 in turn is expressed in renal blood vessels and regulates blood pressure (Kotlo et al., 2020). However, the actions of SCFAs are not limited to the tissues that express SCFA receptors, because SCFAs can enter cells through monocarboxylate transporters.

### Immune responses modified by SCFAs

Among many immune responses modified by SCFAs was a reversal of the inflammatory changes in monocytes and macrophages in the gut induced by the bacterial metabolite lipopolysaccharide (LPS) (Parada Venegas et al., 2019). SCFAs also inhibited the release of various inflammatory cytokines and chemokines, such as NFkB, TNFα, IL6, IL10, CXCL10 (Segain et al., 2000; Nastasi et al., 2015; Li et al., 2018), CXCL8 and CCL20 (Iraporda et al., 2015), and IL18 (Macia et al., 2015). All these studies were done *in vivo* or *in vitro* in the gut, monocyte-derived dendritic cells, or human umbilical vein endothelial cells. In one study, mice were either orally supplemented with acetate or fed high-fiber diet that increased fecal SCFA levels (Macia et al., 2015). Both exposures reduced IL18 levels, although only oral supplementation with the highest acetate concentration was effective. Many of these cytokines and chemokines have been linked to poor ICB response, especially CXCL8 (Davar et al., 2021).

**Figure 2:**
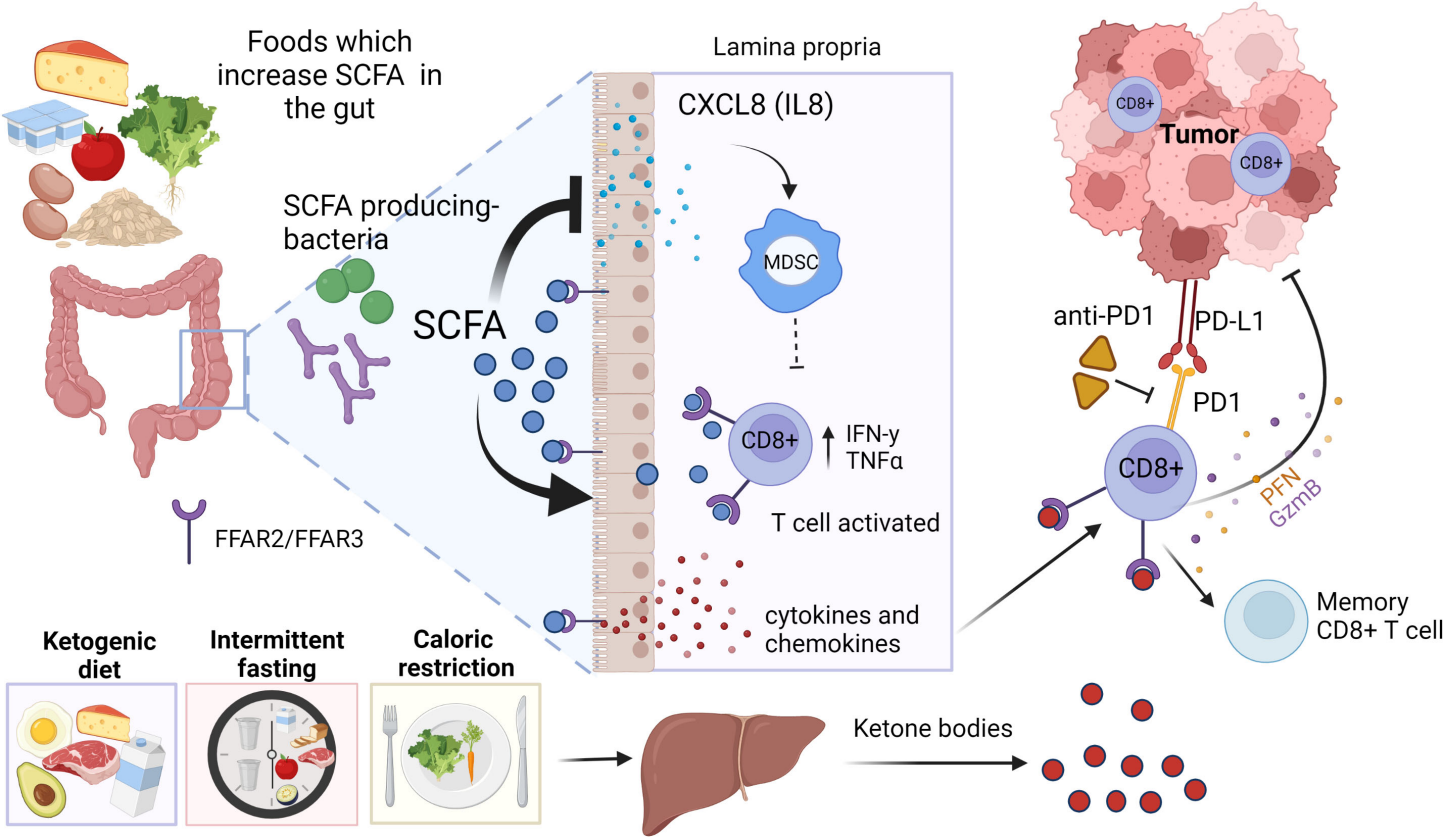
Short chain fatty acids (SCFAs) and ketone bodies as mediators of diet on immune checkpoint blocker (ICB) responsiveness. Foods high in dietary fibers increase the abundance of SCFA producing bacteria, such as *Bifidobacterium species (sp.)* and *Akkermansia (A.) muciniphila*. SCFAs bind to their receptors FFAR2 and FFAR3 in the gut epithelium, immune cells and enteric neurons, releasing cytokines and chemokines that can lead to an activation of CD8+ T cells in the tumor microenvironment (TME). SCFAs also suppress the release of various inflammatory cytokines and chemokines, including CXCL8 which are required for activation of immunosuppressive myeloid derived suppressor cells (MDSCs). Ketogenic diet, fasting and caloric restriction generate ketone bodies in the liver. Ketone bodies can bind and activate FFAR2/FFAR3 in immune cells, resulting activation of CD8+ T cells. Created in Biorender.com.

SCFAs also affect adaptive immune cells in the gut (Correa-Oliveira et al., 2016; Deleu et al., 2021). In a study by Luu et al. (Luu et al., 2021), SCFAs increased the production of CD8+ T cell effector molecules, such as CD25, IFNγ and TNFα, and enhanced the anti-tumor activity of antigen-specific CD8+ T cells in syngeneic murine melanoma and pancreatic cancer models. In this study, immune cells were treated with SCFAs prior they were transferred to Rag1-/- mice, i.e., mice were not treated with SCFAs but only immune cells. Bachem et al. (Bachem et al., 2019) showed that the microbiota-derived butyrate, resulting from feeding mice a high-fiber diet, reversed the inability of antigen-activated CD8^+^ T cells to transition into long-lived memory cells in GF mice. Further, butyrate reversed transcriptional impairments in core genes associated with oxidative metabolism in CD8^+^ T cells in GF mice by leading to uncoupling of the tricarboxylic acid (TCA) cycle from glycolytic input and instead allowing fatty acid catabolism and glutamine utilization to fuel oxidative phosphorylation (OXPHOS). These results indicate that SCFAs can promote CD8^+^ T cells by inducing the metabolic pathways that support their activity and ability to transition to become memory T cells. However, this happened either *in vitro*, or when immune cells were treated with SCFAs before transferring them to mice, or in mice fed butyrate producing high-fiber diet. Thus, the effects of SCFAs on immune cells likely take place already in the gut. **Figure 2** summarizes the effects of SCFAs on cytokines and immune cells.

### SCFAs and ICB response

A study by Nomura et al. (Nomura et al., 2020) assessed a possible link between fecal and plasma levels of SCFAs and responsiveness to PD1 inhibitors among cancer patients with solid tumors. 52 patients in Kyoto, Japan were recruited for the study, and these patients were followed for an average of 2 years. Of the treated patients, 28.8% responded to the therapy; i.e., a percentage typically reported in similar studies. High concentrations of all the three key SCFAs in the feces - acetic acid, propionic acid, and butyric acid - were positively linked to anti-PD1 effectiveness in patients. Two other human studies have assessed a possible link between SCFAs and responsiveness to ICB treatment. The gut metabolomic profile was analyzed in 11 NSCLC patients receiving second-line treatment with anti-PD1 nivolumab (Botticelli et al., 2020). Patients progressing late, i.e., had a good treatment response, exhibited higher fecal levels of SCFAs propionate and butyrate, compared with patients progressing early. Coutzac et al. (Coutzac et al., 2020) assessed a correlation between SCFA producing bacteria and responsiveness of 26 metastatic melanoma patients to anti-CTLA4 therapy. They found that a high abundance of *Faecalibacterium* correlated with longer progression free survival.

Although the data are consistent in linking high abundance of SCFA producing gut bacteria and high fecal SCFA levels on ICB responsiveness, serum SCFA levels or oral SCFA administration has generated contrasting findings. In Coutzac et al. (Coutzac et al., 2020) study, serum butyrate and proprionate levels were associated with shortened progression free survival among metastatic melanoma patients. Serum acetate levels were not linked to impaired ICB response. In the Nomura study (Nomura et al., 2020), circulating SCFAs did not correlate with ICB response either. Botticell et al. (Botticelli et al., 2020) did not determine circulating SCFA levels and ICB responsiveness. There are several factors in play that might contribute to these data. Circulating SCFAs can originate either from a person’s own gut microbial production which is promoted by a consumption of fiber rich foods and a consumption of foods enriched in SCFAs (Wolfe and Dutton, 2015). Fecal SCFA levels correlate with circulating SCFA concentrations (Coutzac et al., 2020; Yamamura et al., 2020), but in some studies circulating butyrate levels have been found to negatively correlate with the abundance of SCFA-producing Firmicutes and *Faecalibacterium prasnizii* in the gut microbiota (Coutzac et al., 2020). Similar to fecal SCFAs of which only very small amounts enter peripheral blood once they reach the liver, SCFAs in foods are rapidly absorbed in the stomach or small intestine and cleared from circulated blood within 1 hr (Pomare et al., 1985; Chambers et al., 2015).

In animal studies, supplementation with butyrate improved effectiveness of anti-PD1 therapy against MC38 colon cancer in one experiment (Zhang et al., 2021), but it did not affect response to anti-PDL1 in the same model in another experiment (Jing et al., 2021b). Adverse effects on to anti-CTLA4 responsiveness were reported in three different cancer models in mice treated with sodium butyrate *via* drinking water (Coutzac et al., 2020). Butyrate supplementation also impaired dendritic cell maturation and T cell priming and increased Treg cell expansion in the study. Further, intratumoral butyrate levels correlated with impaired DC function and CD8+ T cell activation (Coutzac et al., 2020). Other mouse studies have generated data suggesting that oral SCFA administration promotes inflammation (Nadeem et al., 2017; Nakajima et al., 2017). Thus, more research is needed before starting to supplement orally cancer patients treated with ICB with SCFAs. A safer approach is likely to be a consumption of dietary fiber and fermented foods to boost fecal SCFA levels. It is possible that fecal SCFAs improve ICB responsiveness through affecting the gut immune cells and gut enteroendocrine cells rather than directly affecting the immune cells in the TME.

### Inosine

Besides SCFAs, other potential microbial metabolites to mediate the effects of the gut microbiota on immune cells and responsiveness to ICBs include inosine (Mager et al., 2020) and SagA (Griffin et al., 2021). Inosine is a metabolite of, for example, *Bifidobacterium pseudolongum* and *A. muciniphila;* i.e., two bacteria found to promote ICB effectiveness and SCFA production (Routy et al., 2018; Derosa et al., 2020). Mager et al. (Mager et al., 2020) first determined alterations in fecal bacterial composition in mice with azoxymethane (AOM) and dextran sulfate sodium (DSS) -induced colonic tumors that were either treated or not treated with anti-CTLA4 or anti-PD1 antibodies. The most significant change was an increase in the abundance of *Bifidobacterium pseudolongum* in the ICB treated mice. Exposure of mice to this bacterium, or serum from mice treated with *B. pseudolongum*, improved response to ICB. Inosine was then identified as the *B. pseudolongum* metabolite responsible for increasing ICB effectiveness. The investigators further discovered that the ability of inosine to improve ICB response was through activation of CD8+ T cells (Mager et al., 2020).

### SagA

In some studies, the bacterial genus *Enterococcus* is enriched in patients responding to ICBs (Routy et al., 2018; Matson et al., 2018). One of its species, *E. faecium*, has distinctive peptidoglycan composition and remodeling capabilities to enhance host tolerance to enteric pathogens. *Enterococci* species were found to express and secrete orthologs of the peptidoglycan NlpC/p60 hydrolase SagA (Griffin et al., 2021). SagA, in turn, generates immune-active muropeptides that promote responsiveness to anti-PDL1 therapy. The investigators further showed that SagA-engineered probiotics or synthetic muropeptides potentiated ICB efficacy (Griffin et al., 2021).

Since SagA producing *Enterococcus* species also are producers of acetate (Sarantinopoulos et al., 2001; Fujita et al., 2020), and *Bifidobacterium pseudolongum* and *A. muciniphila* that produce inosine are producers of acetate and propionate (Derrien et al., 2004; Riviere et al., 2016; Louis and Flint, 2017), the links observed with inosine or SagA and ICB responsiveness might also reflect increased production of fecal SCFAs.

### Microbial antigen mimicry

Another explanation for the gut microbiota–immune response link could be antigen mimicry between microbes and tumor antigen epitopes. Several investigators have found similarities between tumor neoantigen epitopes and microbial epitopes (Snyder et al., 2014; Fluckiger et al., 2020; Bessell et al., 2020). Fluckiger et al. (Fluckiger et al., 2020) discovered that T cells in cancer patients cross-recognized tumor antigens and microbial antigens. Further, when a specific antigen from a bacteriophage in mice was presented to its CD8^+^ T cells, the cells recognized and attacked tumor cells expressing the same epitope (Fluckiger et al., 2020). In another study, *Bifidobacterium breve* was identified to express an epitope that cross-reacted with B16.SIY melanoma cells (Bessell et al., 2020). These cells were engineered to express the model antigen SIYRYYGL which was recognized by CD8^+^ T cells to improve their immunogenicity. Investigators found that T cells from mice lacking *B*. *breve* were less reactive than from *B*. *breve* –colonized mice, and the T cell response was transferable by co-housing the mice. In addition, B16.SIY tumors grew faster in mice lacking *B*. *breve* (Bessell et al., 2020). Therefore, the gut microbiota antigens may be directly involved in activating T effector cells to eliminate cancer cells.

## Estrogens as modifiers of ICB response

### Estrogens affect the immune cells

Estrogens affect immune cells and thus could impact ICB responsiveness. Many immune cells, including dendritic and myeloid cells, macrophages and T lymphocytes, express the ERα (Rothenberger et al., 2018). Further, through ERα estrogens activate tumor-associated macrophages (Svensson et al., 2015), increase the number and function of immunosuppressive Tregs and Th2 cells (Khan and Ansar, 2015), drive mobilization of MDSCs (Svoronos et al., 2017), and reduce anti-tumor CD8+ T cells (Polanczyk et al., 2006). This perhaps explains the findings that E2 not only is the key tumor growth promoting factor in ERα breast cancer (Pike et al., 1983; Dickson and Lippman, 1987), but E2 administration also significantly increases ERα negative (ERα-) tumor growth (melanoma, TNBC, lung) in ovariectomized syngeneic mice with intact immune system or in nude mice with innate immune cells (Pequeux et al., 2012). Although E2 stimulates the growth of ERα- cancer cells in syngeneic and nude mice, loss of estrogens inhibits ERα- tumor growth only in syngeneic mice (Svoronos et al., 2017). Thus, adaptive immune cells are activated when estrogens are removed, while estrogens activate immunosuppressive immune cells.

In clinical studies, neoadjuvant treatment (treatment given before surgery) of ERα+ breast cancer patients with aromatase inhibitor (AI) letrozole increased infiltration of effector Th1 cells (Dannenfelser et al., 2017) and reduced Treg infiltration in the TME (Generali et al., 2009). Neoadjuvant hormone therapy consisting of either AI anastrozole or antiestrogen fulvestrant significantly increased tumor infiltrating lymphocytes (TILs) in responding breast cancer patients (Liang et al., 2018). An opposite result was obtained in a study in which the increase in TILs during 4-month neoadjuvant letrozole therapy was predictive of poor letrozole response (Skriver et al., 2020). TILs contain both CD8+ T cells and Treg cells, and breast cancer studies that use TIL infiltration as an end-point do not often specify which T cells were studied. In a neoadjuvant trial in which CD8+ T cells and Tregs were analyzed separately, reduced Treg infiltration was predictive of good response to AI therapy (Generali et al., 2009). Thus, in ERα+ breast cancer, neoadjuvant hormone therapy is most effective in patients who respond to this therapy by exhibiting reduced Tregs in the TME.

In many adjuvant studies involving ERα+ patients, high infiltration of either CD8+ T cells and Tregs has been predictive of poor response to the subsequent hormone therapy (Ali et al., 2014; Krishnamurti et al., 2017; Miyoshi et al., 2019). This contrasts the findings obtained in TNBC patients who across studies show better survival if their pre-treatment tumors exhibit high infiltration of CD8+ T cells. Treatments of TNBC do not include hormone therapy. TNBCs harbor higher infiltration of CD8+ T cells than ERα+ breast cancers, including exhausted CD8+ T cells (Egelston et al., 2022). The presence of high levels of exhausted CD8+ T cells predicts longer survival in TNBC but has the opposite effect in premenopausal ERα+ breast cancer patients (Egelston et al., 2022). Dr. Munster’s group has investigated whether T cell infiltration affected response to ICBs among recurring ERα+ breast cancer patients who received hormone therapy (Terranova-Barberio et al., 2020). All patients who exhibited CD8+ T cell exhaustion and treatment-induced depletion of Tregs in tumor or blood showed clinical benefit (Terranova-Barberio et al., 2020). Although many questions remain, ERα+ breast cancer patients who respond to neoadjuvant and adjuvant hormone therapies, including when also receiving ICB, show a reduced Treg infiltration. Thus, treatment-induced growth suppression of ERα+ breast cancers may involve controlling of immunosuppression rather than activation of CD8+ T cells.

### Gender as a factor in ICB response

Men and women exhibit different immune responses. Women have more macrophages, CD4+ and CD8+ T cells and B cells, and upon stimulation these immune cells become activated more than the same cells in men (Schafer et al., 2022). Consequently, women exhibit better vaccine response and generate more antibodies for example against Covid-19 virus than men. Men are more vulnerable than women for developing infectious diseases and many cancers, including bladder, kidney, liver, lung and stomach cancers (Schafer et al., 2022). However, due to their superior effector T cell responses, women more often suffer from autoimmune diseases. Although higher estrogen levels in women may partly explain their better adaptive immune regulation, androgens that are higher in men than women may explain their poorer immune responses (Ben-Batalla et al., 2020; Irelli et al., 2020).

Due to the well-established gender-specific differences in immune responses, it has been studied whether men and women respond differently to ICBs. Several meta-analyses have attempted to clarify the results of individual studies that have been conflicting. In two of the meta-analysis male NSCLC or melanoma patients responded significantly better to ICBs than female patients (Conforti et al., 2018; Conforti et al., 2021), while one did not find any evidence to support a gender-difference in ICB response (Wallis et al., 2019). The most recent meta-analysis found the opposite: women with NSCLC responded significantly better than men to chemo-ICB therapy combination (Takada et al., 2022). It is possible that chemotherapy is more effective in inducing immune cell activation in women than men, and therefore women are more responsive to the combination of chemo- and ICB therapy than men. A well-controlled single study including 103 patients, published in 2022, reported that men with advanced melanoma responded significantly better to immunotherapy than women (Kudura et al., 2022). Although it is not possible to make any solid, evidence-based conclusion from these data, we lean on the side that men with cancer are more responsive to ICBs than women. Whether the higher estrogenicity in premenopausal women or higher androgen levels in men contributes to the potential gender-specific difference in responding to ICB therapy should be studied.

### Estrogens and PD1

Estrogens upregulate PD1 in immune cells and its ligand PDL1 in immune and cancer cells (Polanczyk et al., 2007; Weber et al., 2018; Nam et al., 2019). If availability of ICB receptors, such as PD1, improve the ability of ICBs to activate T cells, estrogens would be expected to improve ICB response. However, high PD1 expression is linked both to CD8+ T cell exhaustion (Ma et al., 2019) and improved CD8+ T cell anti-tumor reactivity (Simon and Labarriere, 2017), making it difficult to predict whether estrogens impair or improve ICB response. Nevertheless, since estrogens suppress CD8+ T cells and upregulate Treg cells, there will be more PD1 expressing Treg than CD8+ T cells in high estrogenic environment. Consequently, when ERα+ breast cancers are treated with ICBs, ICBs result invigoration of Treg cells (Kamada et al., 2019; Tan et al., 2021). There will not be enough CD8+ T cells to be activated by ICBs in a high estrogenic environment to counteract the activation of Treg cells, and consequently, ICBs might cause immunosuppression.

**Figure 3:**
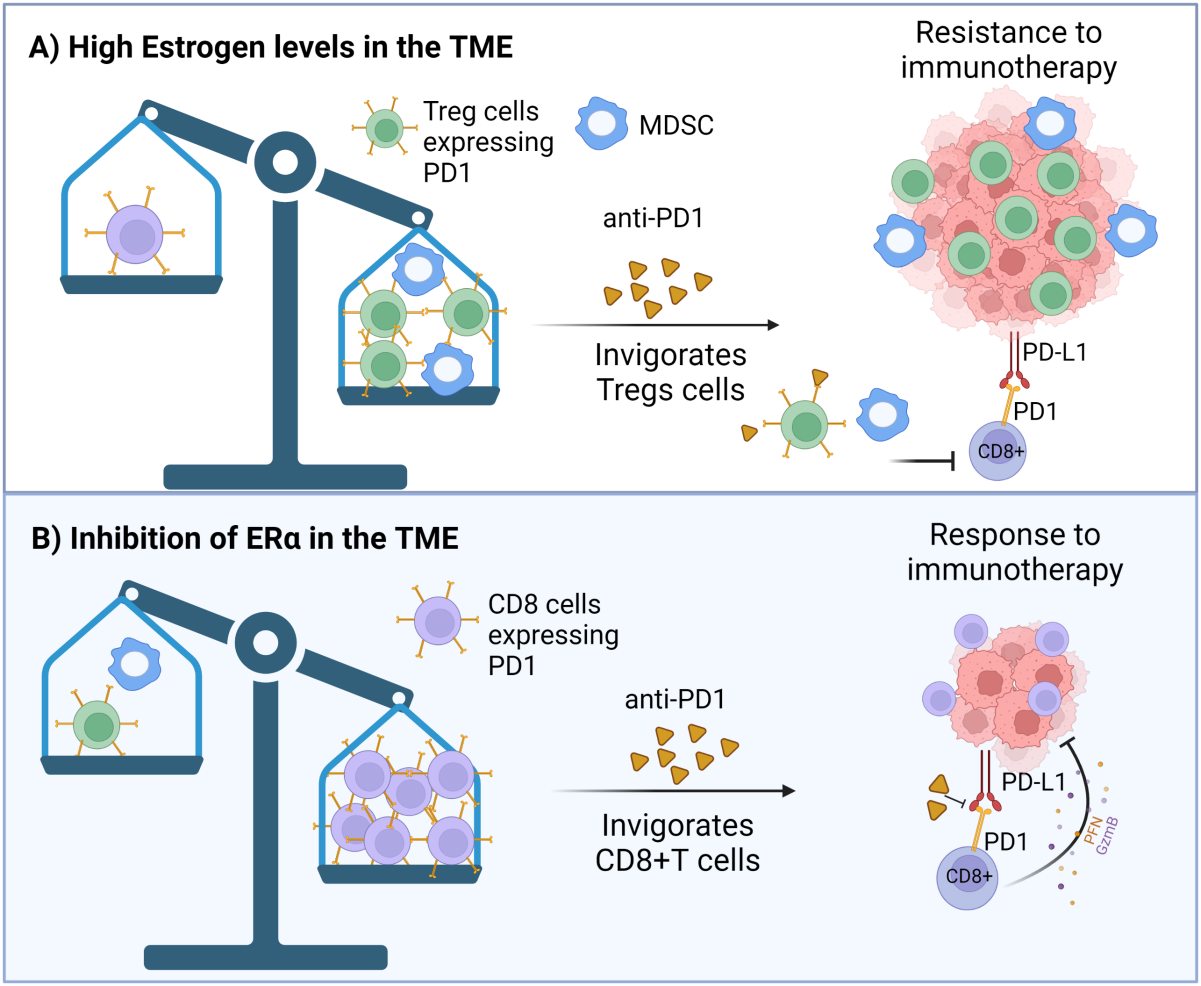
**(A)** Effect of high estrogenic environment, or **(B)** inhibition of the estrogen receptor α (ERα) in immune cells on ICB responsiveness. **(A)** ERα+ breast cancers grow in highly estrogenic tumor microenvironment (TME), while estrogen levels are not elevated in the TME of TNBC. Because estrogens suppress CD8+ T cells and upregulate Treg cells in the TME, and upregulate PD1 expression in both types of immune cells, there will be more PD1 expressing Treg than CD8+ T cells in high estrogenic environment. Consequently, inhibition of PD1 by ICB treatment in ERα+ breast cancers will invigorate Treg cells, leading to ICB immunotherapy resistance. **(B)** If ERα is inhibited in MDSCs by hormone therapy, their activity is inhibited, allowing the number of CD8+ T cells expressing PD1 to be increased and Tregs to be suppressed. Anti-PD1 therapy can then specifically invigorate CD8+ T cells. Created in Biorender.com.

If estrogenicity is inhibited by hormone therapy, the number of CD8+ T cells is found to be increased and Tregs to be suppressed (Skriver et al., 2020; Hazlett et al., 2021). Thus, during hormone therapy, anti-PD1 may invigorate CD8+ T cells. The idea that blocking ERα in immune cells with endocrine therapy will improve responsiveness to ICBs is supported by findings obtained in TNBC in a preclinical model: inhibition of ERα with antiestrogen fulvestrant increased the sensitivity of TNBC 4T1 mammary tumors to PDL1 inhibitor in syngeneic mice (Marquez-Garban et al., 2019). Further, E2 stimulated the growth of ERα- melanoma in mice, and refractory melanoma was converted to be ICB responsive by treating mice with antiestrogen fulvestrant (Chakraborty et al., 2021). **Figure 3** illustrates the interactions among estrogens/ERα, CD8+ T and Treg cells, and ICB response.

In summary, estrogens may be pro-tumorigenic in cancers which do not express ERα by promoting Tregs, MDSCs and Th2 cells, tumor associated macrophages and PDL1, and by increasing the release of IL6, IL17A and TNFα (Rothenberger et al., 2018). These immunosuppressive and tumor growth -promoting changes occur through estrogens binding and activating ERα in the immune cells. Blockage of ERα or suppression of estrogen levels would potentially be antitumorigenic *via* activation of M1 macrophages, Th1 cells and CD8+ T cells as well as increased release of IFNγ and IL12 (Rothenberger et al., 2018). Consequently, inhibition of ERα in immune cells would be expected to improve ICB responsiveness: results from preclinical studies support this conclusion (Marquez-Garban et al., 2019; Chakraborty et al., 2021).

## Estrogen - gut microbiota interaction

Estrogens derived from ovaries, adrenal glands, adipose tissue, and other tissues as well as estrogenic compounds in diet and environment modify the composition of the gut microbiota. In a mouse study, administration of conjugated estrogens and bazedoxifene, a combination that are used to treat menopausal symptoms in women, suppressed the abundance of *Akkermansia* (Chen et al., 2018). This might reduce SCFA production. No other changes in the microbial taxa were observed. In another study, treatment of male mice and ovariectomized female mice with E2 increased alpha-diversity but suppressed F/B ratio (Song et al., 2020). Consistent with this finding, ovariectomy reduced alpha-diversity and SCFA production and increased Bacteroidetes phyla in female mice and rats (Cox-York et al., 2015; Kosaka et al., 2021). Results from studies done in men and postmenopausal women indicated that circulating estrogen levels, which originate from aromatization of androgens in various non-ovarian tissues and are relatively low, correlated with increased alpha-diversity (Flores et al., 2012). In premenopausal women with notably higher circulating estrogens, no correlation between estrogen levels and the three potential markers of ICB responsiveness were seen (Flores et al., 2012). These findings indicate that changes in gut microbial ICB response markers are not consistently linked to circulating estrogen levels.

The ability of some bacterial species to increase GUS activity, and consequently elevate circulating estrogen levels (Baker et al., 2017), might impair ICB response by increasing the ligand availability for hormone receptors in immunosuppressive cells. However, this is unlikely to be the case. The bacteria that produce GUS enzymes include *Lactobacillus, Bifidobacteria*, and *Clostridium* (Kwa et al., 2016), and at the species level *Fecalibacterium prausnitzii*; all these bacteria are SCFA producers and have been linked to improved ICB responsiveness (Sivan et al., 2015; Davar et al., 2021; Lee et al., 2021b; Oh et al., 2021). The levels of GUS are also inversely associated with *Ruminococceae* abundance (Chen et al., 2018), which is consistent with high-fiber diet increasing *Ruminococceae* and reducing GUS levels (Lampe et al., 2002; Tai and Welch, 2005). Thus, most gut microbes that produce GUS also produce SCFAs, and are consistently linked to high effectiveness of ICBs against cancer. Whether the increase in circulating estrogen levels by GUS activity, induced by high abundance of GUS producing bacteria, diminishes the ability of an increase in SCFAs produced by the same bacteria to potentiate ICB responsiveness is not known.

A comparison of the gut microbiota composition between patients diagnosed with ICB refractory ERα breast cancer and ICB responsive TNBC indicated that ERα+ patients exhibited a reduction in the abundance of three genera of *Firmicutes* (*Enterococcus*, *Turicibacter and Veillonella)* and of one *Proteobacteria taxa (Haemophilus*), compared with TNBC patients (Wu et al., 2020). Since *Enterococcus* is one of producers of acetate (Sarantinopoulos et al., 2001; Fujita et al., 2020) and high abundance of *Enterococcus* has been linked to good ICB response (Routy et al., 2018; Matson et al., 2018), it is possible that the gut microbiota composition is involved in ICB refractoriness in ERα+ breast cancers. The origins of the gut microbiota difference might be cancer type itself or factors that determine ERα status in the tumors. However, as circulating estrogen levels are not reported to be different in women who develop ERα+ and TNBC, they are unlikely to explain the difference in the gut microbiota. Taken together, although circulating estrogen levels may modify the gut microbiota, the resulting changes might not impact ICB responsiveness. Nevertheless, circulating estrogen levels may still directly affect immune cells in the TME.

## Estrogenic foods and breast cancer

If high estrogenicity in the TME promotes immunosuppression, it is possible that foods that increase estrogen levels or contain estrogens that activate the ERα might drive poor response to ICBs against any cancer. Foods that contain more than 0.5 ng estrogens/100 g food include butter, chicken, eggs, milk, turkey, wheat and yogurt (Palacios et al., 2020). It has been estimated that a person following USDA healthy eating plan and consuming 2,000 kcal daily obtains up to 0.4 µg food-derived estrogens daily. This amount is unlikely to have any impact even on postmenopausal women whose daily endogenous estrogen production in non-gonadal tissues is about 60 µg. Since endogenous estrogen production per day in men is 100 µg and in premenopausal women 70-700 µg, depending on the stage of the estrus cycle, food-derived estrogens have a very minor impact on total estrogenic load in either women or men.

However, when estrogenicity of different foods is estimated by linking the foods to woman’s circulating estrogen levels, high estrogenic foods increase breast cancer risk. Estrogenicity of foods and breast cancer risk was assessed retrospectively among 37,004 Swedish women (Harris et al., 2015) and prospectively in almost 30,000 women in the USA (Guinter et al., 2018). In the Swedish study, high estrogenic foods were those that were linked to elevated blood E2 or estrone sulfate levels. Using these criteria, the highest estrogenic foods were legumes, pizza and red meat, and the lowest estrogenic foods were coffee and whole grains. The study that was carried out in the USA identified foods as estrogenic if they were associated with elevated circulating E2 levels or resulted reduced ratio between two estrogen metabolites: 2- versus 16-hydroxylated estrogens. 16-hydroxylated estrogens are potentially carcinogenic, whilst 2-hydroxylated estrogens are not (Telang et al., 1992). High estrogenic foods were cheese, cruciferous vegetables, fish/shellfish high in omega-3 polyunsaturated fatty acids (PUFAs), franks/luncheon meats, refined grains (white flour) and tomatoes. Intakes of nuts and seeds, vegetables other than cruciferous vegetables, fish low in omega-3 PUFAs, yogurt and coffee were subtracted to calculate the final food estrogenic score, as these foods were linked to low circulating estrogen levels. In both studies, a dietary pattern associated with high estrogenicity was related to significantly increased breast cancer risk (Harris et al., 2015; Guinter et al., 2018).

Foods identified as being estrogenic in the two studies (Harris et al., 2015; Guinter et al., 2018) do not necessarily contain estrogens, but they might stimulate estrogen production for example by increasing (i) the abundance of gut bacteria which produce GUS or (ii) the conversion of androgens to estrogens by stimulating aromatase. Thus, the increase in breast cancer risk could reflect the ability of estrogens to directly stimulate breast cancer cells and/or impair anti-tumor immune responses *via* activation of ERα. Also, it is possible that estrogenic foods are not causing an elevation in woman’s estrogen levels but that women with high circulating estrogen levels commonly consume these foods. This is supported by the fact that although cruciferous vegetables were identified as high estrogenic foods in a correlation study (Guinter et al., 2018), in an intervention study woman fed broccoli exhibited an increase in the 2:16 hydroxylated estrogen ratio (Fowke et al., 2000), which is indicative of reduced estrogenicity. Further, the causative effects of estrogenic foods on the ICB response have not been directly studied. Some of these estrogenic foods may in fact improve rather than impair ICB responsiveness, such as omega-3 PUFAs (Kelly et al., 2022). The ICB response improving effect of omega-3 PUFAs may be mediated though changes in the composition of the gut microbiota, such as an increase in the abundance of SCFA producing bacteria (Watson et al., 2018k Telle-Hansen et al., 2022). An observational epidemiological study would be helpful in determining if estrogenic foods modify ICB responsiveness.

## Diet as a modifier of ICB response through inducing changes in the gut microbiota and estrogenicity in the TME

### ERβ and GPER

Although TNBCs do not express ERα, many TNBCs express ERβ. The percentile of ERβ positivity in TNBC vary among studies, and it is not clear whether ERβ is protective or predicts poor survival (Sellitto et al., 2020). The factors causing this controversy include uncertainty as to which antibodies are specific to ERβ, and the presence of multiple ERβ isoforms that might have different functions. E2 binds and activates both ERα and ERβ equally, while phytochemicals, such as genistein, preferentially bind to ERβ (Kuiper et al., 1998; Gong et al., 2014).

TNBCs and ERα+ breast cancers also express G protein coupled estrogen receptor (GPER). This receptor was initially identified in the cellular membrane, but it is now clear that it is also expressed in various cellular organs, including mitochondria (Luo and Liu, 2020). Like ERβ, GPER might promote good or poor survival in breast cancer (Hsu et al., 2019). It is also preferentially activated by phytoestrogens (Du et al., 2018; Calfio et al., 2021; Li et al., 2022). In immune cells, activation of ERβ (Zhao et al., 2018; Yuan et al., 2021) and GPER (Natale et al., 2018) can activate CD8+ T cells. In turn, the activation of T cell receptor (TCR) signaling in CD8+ T cells triggers ERβ phosphorylation to further promote TCR signaling cascade and the effectiveness of ICB immunotherapy (Yuan et al., 2021). Further, ERβ agonists reduced tumor MDSC infiltration and enhanced tumor response to ICB therapy (Huang et al., 2020). ERβ knockout mice also exhibited impaired antitumor immunity (Yuan et al., 2021). These results suggest that estrogenic compounds which preferentially activate ERβ or GPER might improve ICB responsiveness in cancer patients.

### Plant-derived phytochemicals (plant estrogens) as modifiers of ICB response

If the composition of the gut microbiota and presence of ligands that preferentially activate ERβ and GPER in the TME can improve ICB responsiveness, the key question is how a cancer patient can acquire ICB responsive gut microbiota as well as activate ERβ or GPER in the TME. FMT from an ICB responsive patient is a promising tool to achieve optimal gut microbiota, but it still poses multiple challenges, especially by its potential to lead to a deadly *E. coli* infection and other adverse effects (Park and Seo, 2021). There are several pharmacological compounds that can activate ERβ and GPER. Nevertheless, dietary factors which can beneficially modify both the gut microbiota and ERβ and GPER activation may be preferable.

Plant-based foods contain high levels of plant-derived estrogens or phytoestrogens: soy contains over 20K µg/100 g, nuts 32K µg/100 g, cereals and bread 1K µg/100 g and olive oil 181 µg/100 g of phytoestrogens (Palacios et al., 2020). Thus, if a premenopausal woman at the highest monthly estrogen producing levels consumes 100 g of nuts daily, she will be exposed to 45 times more phytoestrogens than ovarian estrogens. Perhaps the best-known phytoestrogen in the cancer field is genistein in soy foods: it has a chemical structure similar to E2. However, as already mentioned, genistein preferentially binds to ERβ (Kuiper et al., 1998; Gong et al., 2014), and consequently has different effects on inflammatory cytokines and immune cells than E2. Genistein, in contrast to E2, inhibits master inflammatory response inducer NFkB (Li et al., 2005), and upregulates IFNγ (Guo et al., 2007; Ghaemi et al., 2012) and IL2 (Parry et al., 2009): these changes activate anti-tumor immune responses. In our study, genistein increased the expression of CD8a gene in the mammary tumors in rats also exhibiting improved response to antiestrogen tamoxifen (Zhang et al., 2017a). Further, genistein suppressed Foxp3/Treg expression (Zhang et al., 2017a) and circulating MDSC levels (Lesinski et al., 2015). Genistein’s anti-inflammatory (Du et al., 2018) and metabolism-improving (Li et al., 2022) effects also are mediated through GPER. However, physiological concentrations of genistein suppresses CD4(+) thymocytes in mice; CD8(+) thymocytes were reduced only with pharmacological genistein doses (Yellayi et al., 2002).

Whether genistein reduces or promotes response to hormone therapy in breast cancer remains unresolved. Genistein promoted the growth of MCF-7 human breast cancer cells (ERα+, ERβ-) and *in vivo* tumors, and impaired their response to tamoxifen in nude mice (Allred et al., 2001; Ju et al., 2002). However, breast cancer patients consuming soy foods were at a significantly reduced risk of breast cancer recurrence (Shu et al., 2009; Kang et al., 2010). We found that life-time genistein intake sensitized ERα+ mammary tumors to tamoxifen in rats (Zhang et al., 2017a), while adding genistein to a diet first time when tamoxifen therapy started impaired tamoxifen’s ability to inhibit mammary tumor growth. Importantly, the Global Cancer Update Programme identified soy foods as the only specific food group reducing the risk of breast cancer recurrence; high fiber and vitamin D intake were associated with reduced breast cancer mortality but not recurrence (Tsilidis et al., 2022).

### Phytoestrogenic compounds modify the gut microbiome and ICB response

Genistein modifies the gut microbiota. Mice fed 250 ppm genistein (equivalent of 1 serving of soy foods daily) for 4 weeks exhibited an increased abundance of Verrucomicrobia phylum (Paul et al., 2017). We investigated the ability of 500 ppm genistein supplementation (equivalent of 2 servings of soy foods daily) to modify the tumor immune microenvironment during antiestrogen tamoxifen therapy and to alter the gut microbiome in rats (Andrade et al., 2021). In our study, some of the animals exhibited a persistent gut dysbiosis as a consequence of having been born to an obese dam. Genistein intake resulted in an increase of Verrucomicrobia (Andrade et al., 2021), in agreement with the earlier report in mice (Paul et al., 2017). In addition, the abundance of *A. muciniphila* was significantly increased in genistein-fed animals. Genistein reduced the abundance of pro-inflammatory Proteobacteria phylum (Andrade et al., 2021), which harbor several human pathogens.

In humans, soy milk containing genistein elevated the abundance of *Bifidobacteria*, and fermented soy milk elevated *Bifidobacteria* and *Lactobacilli* and suppressed *Clostridia* (Inoguchi et al., 2012). In another human study, supplementation with 50 mg genistein per day for 2 months led to a significant increase in alpha-diversity and the abundance of *A. muciniphila* (Guevara-Cruz et al., 2020). Further, genistein increased microbial SCFA production (Guadamuro et al., 2017). Since the increased abundance of *A. muciniphila* (Routy et al., 2018; Derosa et al., 2020 #11261) and *Bifidobacteria* (Sivan et al., 2015; Davar et al., 2021; Lee et al., 2021b), and reduced abundance of Proteobacteria (Huang et al., 2021) are linked to improved responsiveness to ICB, genistein might improve tumor response to ICB. We are currently investigating this in a mouse model of TNBC.

Anthocyanins are flavonoids and structurally related to genistein. They are blue, red, or purple polyphenol pigments found in plants, including berries, black rice and red onions. Anthocyanins activate GPER (Calfio et al., 2021), and GPER activation is shown to improve ICB response in pancreatic ductal adenocarcinoma (Natale et al., 2020). Anthocyanins also increase alpha-diversity and fecal SCFA levels (Garcia-Mazcorro et al., 2018; Li et al., 2019; Liu et al., 2020). The ability of anthocyanin in bilberry (this is a wild blueberry growing in Northern Europe which is different from farm-grown blueberries) to impact ICB responsiveness has been explored (Wang et al., 2020; Liu et al., 2020). The results showed that anthocyanin improved responsiveness to anti-PDL1 therapy against MC38 colon tumors in syngeneic mice and enhanced intratumoral CD8+ T cell infiltration. If anthocyanin induced activation of GPER explains the ability of this polyphenol to improve response to ICB therapy, other GPER activating dietary compounds (Khan et al., 2022) may also improve ICB responsiveness.

The low stability and bioavailability of anthocyanins limit their use as health-promoting compounds, and emphasis has been to improve the efficacy and distribution of anthocyanins when ingested (Shen et al., 2022). Interestingly, fertilizing red cabbage with genistein promotes its anthocyanin concentration (Zhang et al., 2016).

Castalagin. There are likely to be several other dietary compounds high in phytochemicals that might improve ICB response. For example, Amazonian berry camu-camu that contains several phytochemicals significantly improved the effectiveness of anti-PD1 against several different cancer types in syngeneic mice, including E0771 TNBC (Messaoudene et al., 2022). The effects were mediated through the gut microbiota and involved activation of CD8+ T cells. Camu-camu increased alpha-diversity and the abundance of *Bifidobacteria* and *A. muciniphila* in the gut microbiota. The study further identified castalagin as the key biologically active compound in camu-camu responsible for improving ICB response (Messaoudene et al., 2022). Castalagin is an ellagitannin found in oak and chestnut wood, and it contributes to the color and the taste of wines and spirits that are stored in oak barrels.

**Table 1** contains all the phytochemicals we were able identify from the literature that have been found to impact the gut microbiota and improve ICB responsiveness in preclinical models. **Supplementary Table 1** provides a list of phytochemical regulated by bacterial enzymes. Cleary, plant derived phytochemicals represent a promising group of compounds that might be developed to both modify the gut microbiota and boost CD8+ T cell activity in the TME and ultimately potentiate effectiveness of ICBs to treat cancer.

**Table 1:** Phytoestrogens that modulate the gut microbiota and affect ICB response in preclinical models.

Phytoestrogen		Gut microbiota changes	Cancer model	Effect on ICB	Reference
Class	Compound				
Stilbenes	Resveratrol	Increased alpha diversity and *Ruminococcus gnavus* and *Akkermansia muciniphila* abundance (Alrafas et al., 2019) Reduced *Enterococcus faecalis*, and increased *Bifidobacterium* and *Lactobacillus* abundance (Shabbir et al., 2021)	Ovarian cancer	Increased mature dendritic cells and cytotoxic T cells Synergistic effect with anti-PD1 antibody	(Zhang et al., 2019)
Flavonols	Quercetin	Reduced Firmicutes/Bacteroidetes ratio and reduced abundance of *Erysipelotrichaceae*, *Bacillus* and *Eubacterium cylindroides* (Etxeberria et al., 2015) Increased abundance of *Fibrobacteres*, *Akkermansia muciniphila*, *Clostridium butyricum*, *Clostridium celatum*, and *Prevotella copri* and decreased abundance of *Proteobacteria*, *Lactobacillus coleohominis*, and *Ruminococcus bromii* (Xu et al., 2021)	Breast cancer	Inhibition of PD1/PDL1 interaction	(Jing et al., 2021a)
Isoflavones	Puerarin	Increased abundance of *Akkermansia muciniphila* (Wang et al., 2019a) Increased diversity of microbiota and abundance of *Lactobacillus*, *Barnesiella*, *Clostridium* IV, *Prevotella* (Mo et al., 2022)	Breast cancer	Increased intra-tumoral infiltration of cytotoxic T cell Synergistic effect with PDL1 blockade therapy	(Xu et al., 2020)
	S-equol	Increased abundance of *Asaccharobacter celatus* and *Slackia isoflavoniconvertens* after daidzein intake (Iino et al., 2019) Increased abundance of *Adlercreutzia equolifaciens* and *Bifdobacterium bifidum* after isoflavone intake (Zheng et al., 2019)	Breast cancer and melanoma	Increased tumor-infiltrating CD8+ T cells Boosted effectiveness of anti-PD1 immunotherapy	(Yuan et al., 2021)
Flavones	Luteolin and apigenin	Increased abundance of *Actinobacteria* (Bian et al., 2020) Increased abundance of *Akkermansia* and *Incertae Sedis* and reduced abundance of *Faecalibaculum* and *Dubosiella* (Qiao et al., 2021)	Lewis lung carcinoma	Synergistic effect with anti-PD1 Reduced expression of PDL1	(Jiang et al., 2021)
Anthocyanins	Bilberry anthocyanin extracts	Increased abundance of *Clostridia* and *Lactobacillus johnsonii* and improved community diversity	Colon adenocarcinoma	Enhanced anti-tumor efficiency of anti-PDL1	(Wang et al., 2020 #11682}
Bilberry anthocyanins	Increased abundance of *Ruminococcaceae* and *Lachnospiraceae*, increased F/B ratio and fecal butyrate levels	Colon adenocarcinoma	Improved therapeutic effects of PDL1; Enhanced intratumoral CD8+ T cell infiltration	(Liu et al., 2020)
Ellagitannin	Castalagin	Increased abundance of *Ruminococcaceae* and *Alistipes*	Sarcoma	Improved CD8+/FOXP3+CD4+ ratio in the TME. Reestablished the efficacy of anti-PD1 therapy in mouse receiving FMT from ICI-refractory NSCLC patients	(Messaoudene et al., 2022)
Other polyphenol compounds with phytoestrogen activity	Curcumin	Increased bacterial richness, inhibition of age-related decrease in alpha diversity, increased abundance of *Lactobacillales*, and decreased *Coriobacterales* order (McFadden et al., 2015) Reduced abundance of *Blautia* spp. and *Ruminococcus* spp. (Peterson et al., 2018) Decreased abundance of *Prevotellaceae* and increased *Bacteroidaceae*, and *Rikenellaceae*. At the genus level, decreased abundance of Prevotella and increased *Alistipes* and *Bacteroides* (Shen et al., 2017)	Breast cancer Colon cancer	Reduced cancer cell expression of PDL1 and sensitized cancer cells to anti-CTLA4 therapy Increased tumor antigen-specific CD8+ T-cell induction, T-cell stimulatory activity of DCs and promoted anti-PDL1 response	(Lim et al., 2016) (Hayakawa et al., 2014)

## Effects of ketogenic diet on ICB response

We will briefly review here other dietary modifications that have been linked to ICB response, possibly by altering the gut microbiota but also by affecting estrogenicity in the TME. These include ketogenic diet, fasting, and caloric restriction. The changes in the gut microbiota caused by ketogenic diet include reduced alpha-diversity and increased abundance of *A. muciniphila* (Olson et al., 2018; Huda et al., 2022). Ketogenic diet has been linked to improved responsiveness to different cancer therapies (Weber et al., 2020). In particular, ketogenic diet is currently being investigated as a potential adjunctive therapy for brain cancers (Seyfried et al., 2011; Thomas and Veznedaroglu, 2020). This diet consists of high levels of fat, moderate levels of protein and low levels of carbohydrates. Ketogenic diet may not elevate circulating estrogens, despite high fat consumption (Molteberg et al., 2022). It has not been studied whether ketogenic diet affects estrogen receptors. A case study reported that ketogenic diet, combined with several alternative therapies, induced a complete and durable response in an end-stage metastatic, ERα+ breast cancer patient (Iyikesici et al., 2021).

Due to low levels of carbohydrates and fiber, ketogenic diet reduces fecal SCFA production (Ferraris et al., 2021). However, ketone bodies, including acetoacetate (AcAc) and 3-beta-hydroxybutyrate (3HB), are generated in the liver when ketogenic diet is consumed. The production of AcAc and 3HB involves enzymatic degradation of fatty acids *via* β-oxidation to form acetyl-CoA in the hepatic mitochondria. Normally, serum acetate levels are higher than AcAc levels, but during ketogenic conditions both AcAc and 3HB are markedly increased, while SCFAs are dropped by several folds (Miyamoto et al., 2019). These ketone bodies then bind to SCFA receptors GPR41, GPR43 and GPR109A (Kimura et al., 2011; Offermanns, 2017; Miyamoto et al., 2019), and can improve anti-tumor immune responses (Hirschberger et al., 2022).

### Ketogenic diet and ICB response

Ferrere et al. (Ferrere et al., 2021) found in a preclinical study that a ketogenic diet inhibited the growth of orthotopic melanoma and renal cancer cells in syngeneic mice. This diet caused an increased abundance of *A. muciniphila*, but led to a loss of more than 10 species of *Lactobacillaceae* family. The study also identified an elevated abundance of *Eisenbergiella massiliensis* (recently identified species of *Clostridia* order under Firmicutes) in ketogenic diet fed mice. An abundance of *Eisenbergiella massiliensis* correlated with a higher presence of ketone bodies in mice and among 1,000 healthy individuals (Ferrere et al., 2021). The tumor-growth inhibiting effect of ketogenic diet was lost in mice treated with an antibiotic mix to deplete the gut microbiota and in mice experimentally depleted of CD4+ and CD8+ T cells. The study also showed that both ketogenic diet and administration of ketone bodies improved the effectiveness of ICBs (anti-CTLA4 and anti-PD1 mAb) against renal cancer in mice (Ferrere et al., 2021). This preclinical study provides preliminary evidence in support of ketogenic diet possibly being beneficial when treating cancer patients with ICBs. Importantly, in contrast to a study in which oral administration of SCFA butyrate *via* drinking water impaired the response to ICB in mice (Coutzac et al., 2020), in this study oral supplementation with 3HB improved ICB responsiveness (Ferrere et al., 2021). Since fecal SCFA levels or high abundance of SCFA producing bacteria are associated with high responsiveness to ICBs, perhaps further improvement might be achieved by combining fiber supplementation with ketogenic diet or other ketonic conditions, such as fasting or physical activity (Evans et al., 2017).

## Caloric restriction and fasting and ICB response

Systematic review and meta-analysis indicated that reduced caloric intake in healthy postmenopausal women reduced total and free estradiol levels (de Roon et al., 2018). Experimental data further suggested that caloric restriction and caloric restriction mimetics (CRM) improved responsiveness to immunogenic death (ICD) inducing chemotherapy (Pietrocola et al., 2016). The mechanism through which CRMs potentiate the ability of chemotherapy to kill cancer cells seems to be mediated through increased autophagy which results in CD8+ T cell activation (Pietrocola et al., 2016). Among the dietary sources of CRM are chrysin in honey, genistein in soy foods, resveratrol in wines, grapes, lingonberry, spermine and spermidine in soy foods, nuts, and seeds, flavan-3-old and quercetin in apples and berries, salicylic acid in berries, fruit juices and wines, and NAD+ precursors in nuts, fish, pork, beef, soy and cheese (Hofer et al., 2021).

Responsiveness to ICB in CRM-treated or fasted mice has also been investigated (Levesque et al., 2019). Mice receiving ICD-inducing chemotherapies responded better to anti-PD1 mAb against fibrosarcoma when either exposed to fasting or CRM (hydroxycitrate or spermidine). However, without chemotherapy, no response to anti-PD1 + fasting/CRM was seen. This is consistent with the clinical evidence that chemotherapy improves ICB responsiveness (Heinhuis et al., 2019). However, Ajona et al. (Ajona et al., 2020) observed improved responsiveness to anti-PD1 by fasting in syngeneic mice allografted lung cancer cells, even when no chemotherapy was given. The difference in the two studies may reflect potential differences in responsiveness of fibroadenoma versus lung cancer to ICB monotherapy.

Caloric restriction affects the gut microbiota, but the effects are inconsistent among studies. Caloric restriction is reported to increase alpha-diversity (Sbierski-Kind et al., 2022) and microbial SCFA production (Zheng et al., 2018). However, in another study caloric restriction reduced fecal SCFAs (Rondanelli et al., 2021) and bacterial diversity, and caused an enrichment in *Clostridioides difficile* (von Schwartzenberg et al., 2021). Since caloric restriction induces a ketogenic condition, it elevates circulating ketone bodies (Miyamoto et al., 2019). Thus, caloric restriction might improve ICB response *via* an increase in ketone bodies and/or by reducing circulating E2 levels and E2 in the TME, both of which lead to improved CD8+ T cell activity (Hirschberger et al., 2022).

One clinical trial is currently investigating the effect of fasting on response to different cancer therapies (Vernieri et al., 2022). Half of the patients have breast cancer, and the most common therapy used is chemotherapy. Only 3 patients received ICB. In the study, fasting lasted for five days, followed by 16 to 23 days of refeeding. Fasting alone, or in combination with standard antitumor therapies, downregulated immunosuppressive myeloid cell subsets, while at the same time increased activated CD8+ T cells (Vernieri et al., 2022). Another study (clinicaltrials.gov: #NCT04387084) is being performed to determine if short-term fasting improves responsiveness to anti-PD1 therapies in patients with advanced melanoma. We are not aware of any clinical studies investigating the potential of CRMs to improve effectiveness of ICBs.

## Obesity: Beneficial effects on the gut microbiota markers of ICB response

Obesity and consumption of a high-fat diet increase circulating estrogen levels by increasing aromatization of androgens in adipose tissues. The increase has been seen in men and postmenopausal women (Schneider et al., 1979; Zumoff et al., 1981; Lukanova et al., 2004). However, in premenopausal women, adipose-derived estrogens suppressed ovarian estrogen production and obese premenopausal women had significantly lower estradiol levels than lean women (Freeman et al., 2010). Obesity also has multiple effects on the gut microbiota, but these effects vary from study to study. Alpha-diversity has been reported to be significantly elevated in obese African Americans (Stanislawski et al., 2019), not to be altered in obese non-Hispanic whites (Stanislawski et al., 2019), and be reduced in a large cohort of obese twins living in the U.K. (Asnicar et al., 2021). Obesity is linked to increased F/B ratio (Turnbaugh et al., 2006; Turnbaugh et al., 2009), and elevated fecal SCFA levels (de la Cuesta-Zuluaga et al., 2018; Kim et al., 2019; Yamamura et al., 2021). The studies showing that obesity increased alpha-diversity, F/B ratio and SCFA levels paradoxically suggest that obesity might improve ICB responsiveness. Experimental data generated in multiple animal models indeed show that obese animals respond better to ICBs than lean animals (Wang et al., 2019b; Cortellini et al., 2019). Improved responses to ICB are also reported in obese cancer patients. Wang et al. (Wang et al., 2019b) assessed progression free and overall survival among 250 patients with lung or ovarian cancer, or melanoma, and found significant improvements in obese patients treated with ICBs, compared with non-obese patients. In addition, obese metastatic RCC patients (Albiges et al., 2016) and melanoma patients (McQuade et al., 2018) were more responsive to ICBs than leaner patients.

Studies are ongoing to understand why obesity improves ICB response. Because of increased fecal SCFA production in obese individuals (de la Cuesta-Zuluaga et al., 2018; Kim et al., 2019; Yamamura et al., 2021), obesity may beneficially impact tumor immune microenvironment when a cancer patient is treated with ICB. Excess body weight induces immune changes both in the adipose tissue and the TME; however, these changes are mostly opposite to each other. In animals fed an obesity-inducing high fat diet, CD8+ T cells influx into white adipose tissue was elevated: this is a classic hallmark of obesity. Leptin, a hormone increased by obesity, seems to be responsible for CD8+ T cell activation under obese conditions (Lord et al., 1998; Naylor and Petri, 2016). Moreover, in adipose tissue leptin polarized naïve CD4+ T cells towards effector Th1 cells with simultaneous inhibition of immunosuppressive Th2 cells (Batra et al., 2010). In contrast to the adipose tissue, obesity impaired infiltration of CD8+ T cells and increased Tregs in the TME (Hillers-Ziemer et al., 2022). However, both in the adipose tissue and the TME, obesity increased the infiltration of MDSC (Ostrand-Rosenberg, 2018). The effect of obesity in impairing CD8+ T cell infiltration to the TME has been demonstrated in many animal models involving allografting syngeneic mice with colon cancer, TNBC and other types of cancers (Gibson et al., 2020; Nunez-Ruiz et al., 2022). In human studies, obese women with ERα+ breast cancers exhibited reduced tumor lymphocyte infiltration, compared with non-obese women (Takada et al., 2021).

One possibility to explain why obesity improves ICB responsiveness, despite suppressing CD8+ T cells in the TME, is that obesity positively impacts immune cell metabolism. Tumor cells compete with immune cells for essential micronutrients (Rodriguez and Ochoa, 2008; Srivastava et al., 2010), and tumor cells exhaust CD8+ T cells by depleting their availability to these nutrients. Lipid uptake in CD8+ T cells is increased in obese individuals, causing fatty acid oxidation to become a more prominent metabolic pathway than glycolysis (Zhang et al., 2020). Enhanced lipid-based metabolism in immune cells has been found to be associated with increased memory generation and decreased CD8+ T cell exhaustion (Zhang et al., 2017b). It is plausible that under obese condition, CD8 +T cells get metabolically reprogrammed and consequently poised to react better to ICB therapy. Importantly, since increased lipid uptake in tumor cells promotes tumor growth, obesity can simultaneously increase cancer risk and mortality, but improve responsiveness to ICB therapy.

Recently, a preclinical model of TNBC (E0771 mammary tumor cells) explored which gut bacteria might be altered in obese mice responding to anti-PD1 (Pingili et al., 2021). Anti-PD1 mAb was found to upregulate *Akkermansia* and *Bifidobacterium* both in obese and lean mice. However, only in obese mice anti-PD1 treatment up-regulated gut microbiota-produced markers of OXPHOS, glycolysis, and pyruvate, arginine, and proline metabolism (Pingili et al., 2021). Whether these bacterial changes contribute to anti-PD1 response has not been studied. Hence, more research is required to define the underlying mechanisms that govern the response to ICBs in obese individuals and the role of gut microbiota in mediating the responsiveness.

## Dietary factors which modify fecal SCFAs, alpha-diversity and F/B ratio: possible link to the ICB response

Below we high-light dietary factors which alter the proposed gut microbiota markers of ICB responsiveness; i.e., SCFAs, alpha-diversity and F/B ratio. Some of them also affect estrogenicity. It is not known whether these diets actually modify ICB response (except fiber and fish oil), but since they are widely consumed by humans, it might be worth performing preclinical and observational epidemiological studies to determine if a connection exists.

### Fiber in plant-foods and ICB response

Plant-based foods high in phytochemicals also contain fiber. Fiber could improve responsiveness to ICB therapy in multiple ways. (i) Fiber-rich foods have been linked to reduced circulating estrogen levels in pre- (Gaskins et al., 2009) and postmenopausal women (Monroe et al., 2007), and this might reduce ERα-mediated activation of immunosuppressive cells in the TME. (ii) Dietary fiber increases alpha-diversity (Rios-Covian et al., 2016; Menni et al., 2017), i.e., one of the markers of ICB responsiveness. (iii) Fiber intake, especially intake of fermented fiber, increases the abundance of SCFA producing bacteria and fecal SCFA levels (Rios-Covian et al., 2016). Dietary fibers are divided to soluble and non-soluble fibers. Examples of soluble fibers are inulin, β-glucan, gums and pectin, and they are present in beans, peas, apples, citrus fruits, oats and barley. Non-soluble fibers can be found in the same foods as soluble fibers. Other good sources of non-soluble fiber are berries, nuts and whole wheat which also contain high levels of phytochemicals. Although it was previously thought that only soluble fibers produce SCFAs, dietary fiber intervention studies have reported similar changes in the gut microbiota composition and SCFA production regardless of the fiber source being soluble or non-soluble (Cronin et al., 2021). In an intervention study in which healthy participants increased their daily fiber intake from 20 g to 40 g, the levels of CAZymes and SCFAs were significantly increased (Wastyk et al., 2021). CAZymes are enzymes that are involved in the formation and break-down of complex carbohydrates and glycoconjugates.

Since plant foods containing fibers increase SCFA production, they might also affect tumor immune responses. A study by Trompette (Trompette et al., 2018) reported that plant foods activated CD8+ T cells, but this study did not involve cancer. Consistent with a high-fiber diet increasing alpha-diversity and boosting CD8+ T cells, results from a correlational study (Spencer et al., 2021) indicate that a high-fiber diet improves the ICB responsiveness. Due to plant-based foods containing phytochemicals and fiber which both beneficially affect the gut microbiota, i.e., upregulate ICB responsiveness markers, and activate ERβ and GPER in immune cells in the TME, there is a need for a clinical intervention study to assess if vegetarian diet will improve response to ICB therapy.

### Alpha-diversity

On top of a list of foods that increase alpha-diversity are plant foods (Reese and Dunn, 2018), including fruits, berries, vegetables, whole grains, seeds, nuts, herbs, coffee and tea. Plant-based foods increase alpha-diversity and Firmicutes levels (Tomova et al., 2019), and reduce circulating estrogen levels (Harmon et al., 2014). Fermentation of plant foods and drinks further increases gut microbial alpha-diversity (Wastyk et al., 2021). Thus, plant foods increase both fecal SCFA production and alpha-diversity. Of individual foods, those high in omega-3 polyunsaturated fatty acids (PUFAs) in fish oil have been linked to increased alpha-diversity (Caesar et al., 2015). Fish oil also has multiple other effects on the gut microbiota, but the changes in the microbial composition regarding phyla or other categories vary from study to study (Costantini et al., 2017). Importantly, fish oil was found to improve immune response of Lewis lung carcinoma and B16F10 melanoma to anti-CTLA4 therapy in mice (Kelly et al., 2022).

Moderate alcohol drinking increases alpha-diversity in humans (Kosnicki et al., 2019) as well as circulating E2 levels by stimulating the aromatase (Purohit, 2000; Rachdaoui and Sarkar, 2013). Perhaps due to alcohol increasing E2 levels, moderate alcohol consumption is cardioprotective (Piano, 2017). Cardiovascular disease (CVD) risk is also causally linked to the gut microbiota (Witkowski et al., 2020). The CVD-promoting gut microbiota composition was not present in moderately alcohol drinking CVD patients, but was seen in heavy drinkers (Zhao et al., 2021). However, pure ethanol in animal studies reduces alpha-diversity (Kosnicki et al., 2019). The difference in the effects of alcohol on the gut microbiota in humans and animal studies likely reflects the fact that alcoholic beverages made of grapes or other fruits, grains and plants (blue agave) contain phytochemicals.

There is a consensus that alcohol increases breast cancer risk (Smith-Warner et al., 1998; Hamajima et al., 2002; Suzuki et al., 2008); however, in specific subgroups this is not the case. These subgroups include (i) African American women (Llanos et al., 2012), (ii) French women consuming regularly 1.5 or fewer alcoholic drinks per day (Bessaoud and Daures, 2008), (iii) postmenopausal Brazilian women who have regularly consumed moderate levels of alcohol for at least 10 years (Vieira et al., 2018), and (iv) postmenopausal U.S. women within a cohort of over 50,000 women who consumed 7 or more drinks per week: their risk of developing TNBC or ERα+ breast cancer was not increased, compared with non-drinkers (Falk et al., 2014). Alcohol affects the immune system. It has been shown that alcohol intake reduces susceptibility to common cold, and improves responsiveness to vaccines (Messaoudi et al., 2014). These immune response improvements are linked to low androgen levels (Ben-Batalla et al., 2020; Irelli et al., 2020), as alcohol intake reduces androgens by stimulating their aromatization (Purohit, 2000; Rachdaoui and Sarkar, 2013). We are not aware of any studies investigating whether alcohol intake impacts ICB response.

Many individuals use products containing artificial sweeteners to reduce daily caloric intake. Consumption of high sugar drinks is shown to increase cancer risk (Makarem et al., 2018), but there is no direct scientific evidence to support the claim that simple sugar intake in foods causes cancer (Laguna et al., 2021). Artificial sweeteners, like saccharin, sucralose, aspartame and neotame modify the gut microbiota composition (Ruiz-Ojeda et al., 2019). For example, relatively new artificial sweetener neotame was reported to reduce alpha-diversity, decrease F/B ratio and decrease bacteria involved in butyrate synthesis in mice (Chi et al., 2018). Also, in mice saccharin has been shown to induce gut dysbiosis and consequently induce liver inflammation (Bian et al., 2017). Sucralose consumption, in turn, caused intestinal inflammation (Rodriguez-Palacios et al., 2018). Consumption of artificial sweeteners is linked to glucose intolerance and type 2 diabetes (Kolodziejczyk et al., 2019), but whether this is causal or correlative is not clear. Findings showing that FMT obtained from artificial sweeter users impaired glucose tolerance in GF host mice suggest that the effects of artificial sweeteners are mediated through the gut microbiota (Suez et al., 2014). It has not been assessed if users of artificial sweeteners might be at high risk of refractoriness to immune therapies.

### Firmicutes to Bacteroidetes ratio

F/B ratio has been used as an indicator of the effect of diet on the gut microbiota. It was initially thought that individuals consuming foods high in fiber and vegetables and low in meats had a low F/B ratio (De Filippo et al., 2010; Jain et al., 2018), whilst obese individuals had a high ratio (Verdam et al., 2013), i.e., Bacteroidetes appeared to be the “healthier” members of the gut microbiota. This idea, largely based on correlational findings, has been challenged by studies showing that individuals consuming a Western diet have a lower F/B ratio than those consuming a plant-based diet (Lin et al., 2013; Szczyrek et al., 2021). Further, a study in which participants were asked to increase their fiber intake and avoid consuming Western foods, led the participants to have an increased F/B ratio (Klimenko et al., 2018). Findings from these studies are consistent with the data showing that high-fiber foods (Spencer et al., 2021) and high F/B ratio (Oh et al., 2021) are associated with improved ICB responsiveness. Thus, plant foods high in fiber positively affect three gut microbial markers linked to ICB response, i.e., high F/B ratio, high SCFA production and high alpha-diversity.

Across multiple studies in mice, depletion of vitamin D3 (VD3) from the diet leads to an increase in the abundance of Bacteroidetes (Waterhouse et al., 2018). Human studies have not generated a consistent pattern of changes in the gut microbiota linked to low VD3 levels (Luthold et al., 2017), and beneficial changes (increased alpha-diversity and *A. muciniphila* levels) in the gut microbiota composition have been reported in some of these studies (Singh et al., 2020). Loss of vitamin D receptor (VDR) in mice and VDR loci in humans is linked to multiple changes in the gut microbiota and its metabolites (Wang et al., 2016). It has been suggested that VD3 supplementation, especially in VD3 deficient people, might improve responsiveness to ICBs (Szczyrek et al., 2021). There is no direct data to support this suggestion.

VD3 has multiple effects on immune cells. It both boosted anti-tumor immunity by suppressing MDSCs (Calvert et al., 2017) and inhibited autoimmune responses by increasing Tregs (Chambers and Hawrylowicz, 2011; Hayes et al., 2015). High tumor VDR expression was associated with upregulation of pathways mediating antitumor immunity and corresponding with higher imputed immune cell scores and histologically detected TILs (Muralidhar et al., 2019). VD3 increased the expression of PD1 in T cells harvested from VD3-treated Crohn’s disease patients (Bendix et al., 2017). VD3-induced changes in immune cells and VD3 depletion-mediated increase in the abundance of Bacteroidetes which are predictive of poor response to ICB in patients (Matson et al., 2018; Oh et al., 2021), suggest that VD3 supplementation might improve ICB responsiveness in some cancer patients.

Because F/B ratio and its potential link to health remains unclear, perhaps mainly due to obesity increasing this ratio, it is not a popular endpoint to assess, even in the context of ICB response. We believe that high F/B ratio reflects high abundance of gut microbial bacteria that produce SCFAs, and fecal SCFAs may be a sufficient marker of ICB response without F/B ratio.

**Figure 4:**
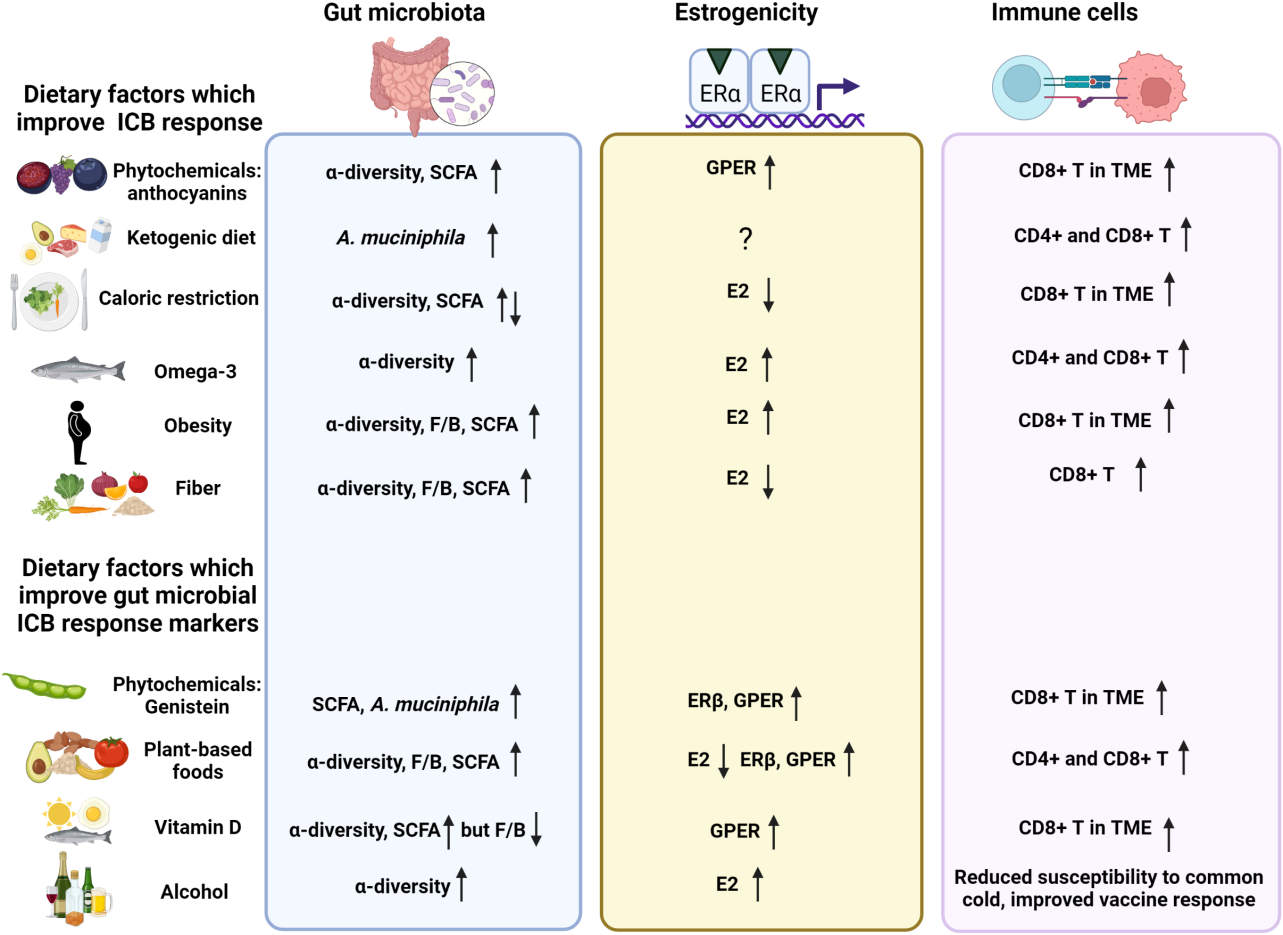
Dietary factors which improve ICB response, or may improve ICB response because they improve microbial ICB response markers. Effects of various dietary factors known to improve ICB responsiveness or which potentially improve ICB responses on the gut microbiota, estrogenicity and activation of CD8+ T cells. Created in Biorender.com.

In summary, many dietary factors might improve ICB responsiveness, such as plant-based foods containing fiber, phytochemicals and plant omega-3 alpha linolenic acids (e.g., nuts, flaxseed, soybeans, avocado). Since artificial sweeteners cause gut dysbiosis, and many individuals use them instead of sugar, observational epidemiological study needs to be performed to determine if artificial sweeteners might cause ICB refractoriness. Finally, because alcohol - cancer connection is complex but alcohol consumption is common among cancer survivors (Sanford et al., 2020), it would be of interest to study if alcohol intake impacts ICB response for example in TNBC patients. **Figure 4** summarizes the effects of various dietary factors discussed above on the gut microbiota, including on markers of ICB response, immune cells and estrogenicity, and ICB response.

## Conclusions

The existing data strongly support a causal connection between the gut microbiota and responsiveness to cancer immunotherapies. The two key observations are: (i) ICB-refractory cancer patients can become responsive after receiving FMT from an ICB-responding donor (Davar et al., 2021; Baruch et al., 2021), and (ii) antibiotics that induce gut dysbiosis and eliminate critical commensal bacteria impair the effectiveness of ICBs (Huang et al., 2019; Yu et al., 2021; Tsikala-Vafea et al., 2021). However, although treatment of mice with probiotics, such as *Bifidobacterium* (Lee et al., 2021b) or *A. muciniphila* (Derosa et al., 2020), improves responsiveness to ICB therapy, findings in humans suggest caution towards using probiotic supplements during ICB therapy (Spencer et al., 2021). The reason why the results from animal studies using probiotics may not translate to humans is that mice originating from the same vendor, consuming the same diet and living in the same environment have relatively similar gut microbiota, while the gut microbiota composition in humans exhibits marked inter-individual differences (Rinninella et al., 2019). Consequently, attempting to modify the gut microbiota of ICB refractory patients towards responsiveness by a single or even multiple probiotics may fail.

Similar to probiotics, foods that positive affect gut microbiota markers of ICB response, may not always be able to reverse ICB refractoriness in cancer patients. This is because genetics influence how the gut microbiota in each individual responds to the foods they eat. In human studies, the role of genetics in impacting the composition of the gut microbiota is less than 10%. Findings obtained by analyzing the gut microbiota in a cohort of 1,126 twins indicated that only 8.8% of the gut microbiota composition can be explain by genetics (Goodrich et al., 2016). However, results from studies in mice suggest that genetics determines how diet modifies the gut microbiota. In the study by Huda et al. (Huda et al., 2022), four different strains of mice exhibited very different changes in the gut microbiota composition when they were fed either Western, Mediterranean, Japanese or Ketogenic diet. Findings from this mouse study indicated that genetics was the main factor explaining the effect of diet on the gut microbiota. Whether it is genetics or factors operating before a child turns 3 years of age and the life-long gut microbiota composition is established, it is clear that the same dietary factor can have completely opposing effect on the gut microbiota of two individuals, or even of the same individual, depending when during the day a dietary factor is consumed (Zheng et al., 2020).

Keeping in mind all the limitations of diets in affecting the gut microbial composition, those that may improve ICB responsiveness are diets/dietary components that increase fecal production of SCFAs or production of ketone bodies in the liver. Plant-derived foods high in fiber, alpha linolenic acid and phytochemicals increase fecal bacteria that produce SCFAs, and fecal SCFA levels. An intervention study (Wastyk et al., 2021) has established that increasing dietary fiber content increased microbial production of SCFAs and a diet supplemented with fermented foods, including fermented plants, increased gut microbial alpha-diversity and suppress circulating inflammatory cytokines. Phytochemicals genistein in soy foods, anthocyanins in for example bilberries, and castalagin in wines and spirits that are stored in oak barrels also increase alpha-diversity and promote SCFA production (Guadamuro et al., 2017; Paul et al., 2017; Garcia-Mazcorro et al., 2018; Li et al., 2019; Guevara-Cruz et al., 2020; Liu et al., 2020; Messaoudene et al., 2022). It is possible that consumption of meat and dairy products obtained from organically maintained, grass-fed farm animals beneficially affect the gut microbiota: these products contain significantly higher levels of phytochemicals than products from traditionally fed farm animals (van Vliet et al., 2021).

Due to the new evidence that oral SCFA administration might adversely affect immune responses in the TME and impair ICB response (Coutzac et al., 2020), SCFAs should not be used as supplements in patients receiving ICBs. However, solid evidence suggests that increased abundance of bacteria which produce SCFAs and high fecal SCFA levels increase ICB effectiveness. We propose that the reason why fecal SCFA levels are beneficial but circulating levels are not is because fecal SCFAs act locally in the gut to improve gut immune responses. How the gut SCFA actions will end up activating CD8+ T cells in the TME remains to be established. In addition to fecal SCFA production, an increase ketone bodies in the liver, as a result of ketogenic condition caused for example by ketogenic diet or fasting, might be most effective method to increase the proportion of patients who respond to ICB therapy. Ketone bodies bind and activate SCFA receptors in immune cells, resulting activation of CD8+ T cells (Polanczyk et al., 2006; Hirschberger et al., 2022).

A question that remains unanswered is whether estrogenic foods which increase circulating estrogen levels or directly activate ERα in immune cells will impair the ICB response. When assessing the effects of estrogens and estrogenic foods on the gut microbiota, the findings indicating that loss of estrogens suppresses gut microbial ICB response markers (Cox-York et al., 2015; Kosaka et al., 2021) and E2 administration reverses these changes (Song et al., 2020) do not necessarily translate to mean that E2 administration improves effectiveness of ICB therapy. Instead, estrogens may impair ICB responsiveness through inhibiting anti-tumor immune cells *via* ERα that is expressed in MDSCs (Svoronos et al., 2017). Findings hinting that men may be more responsive to ICBs than women (Conforti et al., 2018; Conforti et al., 2021; Kudura et al., 2022) further support estrogens in suppressing ICB effectiveness. Phytoestrogens, in turn, might improve anti-tumor immune responses by activating ERβ (Yuan et al., 2021) and GPER (Wang et al., 2020; Liu et al., 2020) in CD8+ T cells, and consequently promote responsiveness to ICBs. Genistein in soy foods preferentially binds and activates ERβ (Kuiper et al., 1998; Gong et al., 2014) and GPER (Calfio et al., 2021) resulting invigoration of CD8+ T cells (Zhao et al., 2018; Natale et al., 2018; Yuan et al., 2021). Anthocyanins from bilberries activate GPER (Calfio et al., 2021). In fact, anthocyanins promote ICB responsiveness (Wang et al., 2020; Liu et al., 2020). These compounds also impact the gut microbiota in a manner that promotes production of SCFAs.

More research is needed to determine if diet-induced changes in the gut microbiota, such as increased production of fecal SCFAs, together with increased production of ketone bodies and activation of ERβ and GPER in the immune cells in the TME, improve effectiveness of ICBs to eliminate cancer. Few trials to investigate if phytochemical in foods affect ICB response have already been started: NSCLC patients receiving anti-PD1 pembrolizumab are supplemented with fermented soybean extract in a study performed in Taiwan (NCT04909034). All clinical trials investigating if foods/diet improves effectiveness of ICBs in cancer patients are listed in **Table 2**.

**Table 2:** Clinical trials involving dietary intervention and ICB to treat cancer.

**Dietary intervention**	**Additional details (when available)**	**NCT**	**Therapeutic intervention**	**Cancer type**	**Phase**	**Status**
Short term starvation	From 24 h before to 24 h after chemotherapy	NCT02379585	Doxorubicin, cyclophosphamide, paclitaxel, docetaxel, trastuzumab, pertuzumab	Breast	Phase 1/2	Terminated, has results
Fasting mimicking diet	For 5 days	NCT03595540	Chemo-, hormone-, targeted or immunotherapies; Opdivo, Keytruda	Breast Colorectal	Pilot, single arm prospective trial	Recruiting
Fasting mimicking diet	From 72 h before to 24 h after chemo +immunotherapy	NCT03700437	Carboplatin/pemetrexed and pembrolizumab	NSCLC		Not yet recruiting
Modified Atkins diet		NCT02768389	Bevacizumab (anti-angiogenic)	Glioblastoma	Early Phase 1	Active, not recruiting
Low protein diet	From 1 week before to 10 days after treatment	NCT03329742	Sipuleucel-T	Metastatic castrate-resistant pancreatic cancer		Recruiting
ACS recommended diet + high fiber	For 11 weeks	NCT04645680	Immunotherapy (pembro + Nivo) as part of standard therapy	Melanoma	Phase 2	Recruiting
Fecal microbiota capsules prepared from one donor who consumed high fiber diet and had high fecal abundance of SCFA producing bacteria (*F. prausnitzii, B. longum, A. muciniphila, Fusobacterium* spp.)		NCT04924374	Anti-PD1 therapy every 2-3 weeks	Advanced Lung Cancer		Recruiting
*Lindera obtusiloba* extract	The safety and efficacy of Lindera obtusiloba on quality of life of cancer patients who are receiving PD1 or PDL1 blockers.	NCT04348149	PD1 or PDL1 blockers	NSCLC		Unknown
The Gut Microbiome and ICB Therapy in Solid Tumors (PARADIGM)	No dietary intervention, but data on diet during intervention available	NCT05037825	Anti-PD1, anti-PDL1, or anti-CTLA4 as a single agent or in combination	NSCLC, Malignant Melanoma, RCC, TNBC		Recruiting
Microbiome in Immunotherapy MIP_NSCLC trial	No dietary intervention, but data on diet during intervention available	NCT04636775	PD1/PDL1 Blockade	NSCLC	Phase 4	Recruiting
Low protein diet	Control diet (~20% protein content) vs low-protein diet intervention (10% protein content)	NCT05356182	PD1, PDL1, CTLA4 inhibitors	Solid tumor malignancies		Recruiting
Immunonutrition; Patients will receive two servings of an oral high-calorie-high-protein nutritional liquid supplement enriched in immunonutrients (Oral Impact^®^)	The intervention will start approximately two weeks before anticancer treatment initiation and will continue up to first disease re-assessment (12-14 weeks) and prolonged according to patient’s needs	NCT05384873	ICBs	Metastatic NSCLC		Not yet recruiting
Fermented soybean extract MicrSoy-20(MS-20), which uses a variety of lactic acid bacteria and yeasts to metabolize organic soybeans	Oral Solution 8 c.c per day divided twice daily (BID) for 48 weeks.	NCT04909034	Pembrolizumab (anti-PD1)	Stage IV NSCLC	Phase 2	Recruiting
Potato starch (Bob’s Red Mill^®^)	Starting 5-7 days before treatment with dual-ICB, participant will consume 20g of potato starch once a day for 3 days, then increase to 20g twice a day, continuing throughout dual-ICB treatment (total duration approximately 13 weeks).	NCT04552418	Ipilimumab and nivolumab in any dose combination (anti-PD1+anti-CTLA4)	Solid tumor	Phase 1	Suspended

## Author contributions

LH-C, wrote the first draft and final version of the manuscript; VV, edited the content of the manuscript and contributed writing sections related to immune checkpoint inhibitors; MM, edited the content of the manuscript and corrected grammar; PK, edited the content of the manuscript and corrected grammar; FA, contributed writing the gut microbiota and diet sections, prepared all figures, edited the content of the manuscript. All authors contributed to the article and approved the submitted version.

## Supplementary material

Supplementary Table 1 Bacterial enzymes that can metabolize phytoestrogens.
