# Supplementary material for: Foods may modify responsiveness to cancer immune checkpoint blockers by altering both the gut microbiota and activation of estrogen receptors in immune cells
Source: Front Microbiomes. 2022 Dec 12;1:1049688. doi: 10.3389/frmbi.2022.1049688 (PMC12993472; doi:10.3389/frmbi.2022.1049688)
Supplement: Supplementary Table 1 — Bacterial enzymes that can metabolize phytoestrogens. [file Table_1.docx]

**Supplementary table 1**: Bacterial enzymes that can metabolize phytoestrogens

| **Phytoestrogen associated with improved ICB response** | **Metabolizing gut bacteria** | **Bacterial metabolic enzyme** | **References** |
| --- | --- | --- | --- |
| **Daidzein and Genistein** | *Escherichia coli* HGH21  Gram-positive strain HGH6  *Bifidobacterium animalis*  *Bifidobacterium longum-a*  *Bifidobacterium pseudolongum*  *Bifidobacterium adolescentis*  *Bifidobacterium breve*  *Bifidobacterium pseudocatenulatum*  *Enterococcus faecalis*  *Lactobacillus acidophilus*  [*Lactobacillus*](https://www.amazon.com/Probiotics-Women-Clinically-Greenselect-Prebiotics/dp/B07PN7FB1R) *gasseri*   Lactobacillus *paracasei* subsp. *Paracasei*  *Streptococcus bovis*  *Bacteroides distasonis*  **Bacteroides** *fragilis*  **Bacteroides** *ovatus*  Clostridium *ramosum*  *Peptostreptococcus productus*  *Bacteroides thetaiotaomicron* | β-glucosidase | (1–3) |
| **Equol** | *Adlercreutzia equolifaciens* is FJC-B9  *Eggerthella* sp.,  *Slackia isoflavoniconvertens*  *Asaccharobacter calatus*  *Enterorhabdus musicola*  *Lactobacillus sp*. Niu-O16 *Eggerthella* sp. Julong 732  *Adlercreutzia equolifaciens*  *Asaccharobacter celatus*  *Eggerthella* sp*.* YY7918  *Eggerthella* sp. Julong 732  Strain DZE  *Slackia isoflavoniconvertens* HE8 | Daidzein reductase  Dihydrodaidzein  Reductase  Tetrahydrodaidzein reductase | (4–12) |
| **Puerarin** | *Dorea* species PUE  *Eubacterium rectale* A-44  *Streptococcus faecium* S-9 *Bifidobacteirum longum* H-1 | *C*-deglycosylation enzymes (DgpA, DgpB-DgpC complex) | (11,13,14) |
| **Resveratrol** | *Lactobacillus  casei*  *Lactobacillus plantarum*  *Lactobacillus acidophilus*  *Aspergillus oryzae sp. 100*  Escherichia coli ATCC 25922,  Bacillus cereus NCTR-466  Achromobacter denitrificans NCTR-774  Slackia equolifaciens  Eggerthella lenta ATCC 4305 | β-Glucosidase  piceid-β-D-glucosidase  *O*-demethylase | (15,16)  (17-19) |
| **Quercetin** | Bacteroides sp. 45  Bacteroides uniformis,  Bacteroides ovatus  Enterococcus avium  Parabacteroides distasonis  *Lachnospiraceae*  *Enterobacteriaceae*  Tannerellaceae  Erysipelotricaceae  Eubacterium ramulus  Enterococcus casseliflavus | α-L-rhamnosidase  β-glucosidase  β-rutinosidase  Flavone reductase | (18,20) |
| **Luteolin and apigenin** | Eubacterium cellulosolvens ATCC 43171  Eubacterium ramulus | β-glucosidase | (18,21,22) |
| **Anthocyanins** | Streptococcus thermophilus GIM 1.*321*  *Lactobacillus plantarum* GIM 1.35  Bifidobacterium spp.  Lactobacillus spp | β-glucosidase | (23,24) |
| **Curcumin** | Blautia sp. MRG-PMF1  *Escherichia coli*  *Escherichia fergusonii* ATCC 35469  *Escherichia coli strains* (ATCC 8739 and DH10B) | *O*-Methyltransferase  NADPH-dependent curcumin/dihydrocurcumin reductase" (CurA) | (25–27) |
| **Ellagitannin** | [Bifidobacterium pseudocatenulatum](https://www.sciencedirect.com/topics/biochemistry-genetics-and-molecular-biology/bifidobacterium-pseudocatenulatum) INIA P815  *Gordonibacter urolithinfaciens* DSM 27213T  *Gordonibacter pamelaeae* DSM 19378  Ellagibacter isourolithinifaciens | Lactonase  Decarboxylase  Catechol-dehydroxylases | (28–31) |

References

1. Hur HG, Lay JO, Beger RD, Freeman JP, Rafii F. Isolation of human intestinal bacteria metabolizing the natural isoflavone glycosides daidzin and genistin. Arch Microbiol [Internet]. 2000 [cited 2022 Nov 18];174(6):422–8. Available from: https://pubmed.ncbi.nlm.nih.gov/11195098/

2. Tsangalis D, Ashton JF, Mcgill AEJ, Shah NP. Enzymic transformation of isoflavone phytoestrogens in soymilk by β-glucosidase-producing bifidobacteria. J Food Sci. 2002;67(8):3104–13.

3. Tsuchihashi R, Sakamoto S, Kodera M, Nohara T, Kinjo J. Microbial metabolism of soy isoflavones by human intestinal bacterial strains. J Nat Med [Internet]. 2008 Oct [cited 2022 Nov 18];62(4):456–60. Available from: https://pubmed.ncbi.nlm.nih.gov/18648905/

4. Maruo T, Sakamoto M, Ito C, Toda T, Benno Y. Adlercreutzia equolifaciens gen. nov., sp. nov., an equol-producing bacterium isolated from human faeces, and emended description of the genus Eggerthella. Int J Syst Evol Microbiol [Internet]. 2008 May [cited 2022 Nov 18];58(Pt 5):1221–7. Available from: https://pubmed.ncbi.nlm.nih.gov/18450717/

5. Cady N, Peterson SR, Freedman SN, Mangalam AK. Beyond Metabolism: The Complex Interplay Between Dietary Phytoestrogens, Gut Bacteria, and Cells of Nervous and Immune Systems. Front Neurol [Internet]. 2020 Mar 13 [cited 2022 Nov 18];11. Available from: https://pubmed.ncbi.nlm.nih.gov/32231636/

6. Tamura M, Hori S, Nakagawa H, Yamauchi S, Sugahara T. Effects of an equol-producing bacterium isolated from human faeces on isoflavone and lignan metabolism in mice. J Sci Food Agric [Internet]. 2016 Jul 1 [cited 2022 Nov 18];96(9):3126–32. Available from: https://pubmed.ncbi.nlm.nih.gov/26455424/

7. Wang XL, Kim HJ, Kang S Il, Kim S Il, Hur HG. Production of phytoestrogen S-equol from daidzein in mixed culture of two anaerobic bacteria. Arch Microbiol [Internet]. 2007 Feb [cited 2022 Nov 18];187(2):155–60. Available from: https://pubmed.ncbi.nlm.nih.gov/17109177/

8. Minamida K, Ota K, Nishimukai M, Tanaka M, Abe A, Sone T, et al. Asaccharobacter celatus gen. nov., sp. nov., isolated from rat caecum. Int J Syst Evol Microbiol [Internet]. 2008 May [cited 2022 Nov 18];58(Pt 5):1238–40. Available from: https://pubmed.ncbi.nlm.nih.gov/18450720/

9. Yokoyama SI, Suzuki T. Isolation and characterization of a novel equol-producing bacterium from human feces. Biosci Biotechnol Biochem [Internet]. 2008 [cited 2022 Nov 18];72(10):2660–6. Available from: https://pubmed.ncbi.nlm.nih.gov/18838805/

10. Wang XL, Hur HG, Lee JH, Kim KT, Kim S Il. Enantioselective synthesis of S-equol from dihydrodaidzein by a newly isolated anaerobic human intestinal bacterium. Appl Environ Microbiol [Internet]. 2005 Jan [cited 2022 Nov 18];71(1):214–9. Available from: https://pubmed.ncbi.nlm.nih.gov/15640190/

11. Jin JS, Nishihata T, Kakiuchi N, Hattori M. Biotransformation of C-glucosylisoflavone puerarin to estrogenic (3S)-equol in co-culture of two human intestinal bacteria. Biol Pharm Bull [Internet]. 2008 Aug [cited 2022 Nov 18];31(8):1621–5. Available from: https://pubmed.ncbi.nlm.nih.gov/18670101/

12. Matthies A, Blaut M, Braune A. Isolation of a human intestinal bacterium capable of daidzein and genistein conversion. Appl Environ Microbiol [Internet]. 2009 Mar [cited 2022 Nov 18];75(6):1740–4. Available from: https://pubmed.ncbi.nlm.nih.gov/19139227/

13. Park EK, Shin J, Bae EA, Lee YC, Kim DH. Intestinal bacteria activate estrogenic effect of main constituents puerarin and daidzin of Pueraria thunbergiana. Biol Pharm Bull [Internet]. 2006 Dec [cited 2022 Nov 18];29(12):2432–5. Available from: https://pubmed.ncbi.nlm.nih.gov/17142977/

14. Nakamura K, Zhu S, Komatsu K, Hattori M, Iwashima M. Deglycosylation of the Isoflavone C-Glucoside Puerarin by a Combination of Two Recombinant Bacterial Enzymes and 3-Oxo-Glucose. Appl Environ Microbiol [Internet]. 2020 Jul 1 [cited 2022 Nov 18];86(14). Available from: https://pubmed.ncbi.nlm.nih.gov/32385077/

15. Basholli-Salihu M, Schuster R, Mulla D, Praznik W, Viernstein H, Mueller M. Bioconversion of piceid to resveratrol by selected probiotic cell extracts. Bioprocess Biosyst Eng [Internet]. 2016 Dec 1 [cited 2022 Nov 18];39(12):1879–85. Available from: https://pubmed.ncbi.nlm.nih.gov/27497981/

16. Chen M, Li D, Gao Z, Zhang C. Enzymatic transformation of polydatin to resveratrol by piceid-β-D-glucosidase from Aspergillus oryzae. Bioprocess Biosyst Eng [Internet]. 2014 [cited 2022 Nov 18];37(7):1411–6. Available from: https://pubmed.ncbi.nlm.nih.gov/24362562/

17. Jung CM, Heinze TM, Schnackenberg LK, Mullis LB, Elkins SA, Elkins CA, et al. Interaction of dietary resveratrol with animal-associated bacteria. FEMS Microbiol Lett [Internet]. 2009 Aug [cited 2022 Nov 18];297(2):266–73. Available from: https://pubmed.ncbi.nlm.nih.gov/19566680/

18. Zhao Y, Zhong X, Yan J, Sun C, Zhao X, Wang X. Potential roles of gut microbes in biotransformation of natural products: An overview. Front Microbiol [Internet]. 2022 Sep 29 [cited 2022 Nov 18];13. Available from: https://pubmed.ncbi.nlm.nih.gov/36246222/

19. Bode LM, Bunzel D, Huch M, Cho GS, Ruhland D, Bunzel M, et al. In vivo and in vitro metabolism of trans-resveratrol by human gut microbiota. Am J Clin Nutr [Internet]. 2013 Feb 1 [cited 2022 Nov 18];97(2):295–309. Available from: https://pubmed.ncbi.nlm.nih.gov/23283496/

20. Riva A, Kolimár D, Spittler A, Wisgrill L, Herbold CW, Abrankó L, et al. Conversion of Rutin, a Prevalent Dietary Flavonol, by the Human Gut Microbiota. Front Microbiol [Internet]. 2020 Dec 21 [cited 2022 Nov 18];11. Available from: https://pubmed.ncbi.nlm.nih.gov/33408702/

21. Braune A, Blaut M. Intestinal bacterium Eubacterium cellulosolvens deglycosylates flavonoid C- and O-glucosides. Appl Environ Microbiol [Internet]. 2012 Nov [cited 2022 Nov 18];78(22):8151–3. Available from: https://pubmed.ncbi.nlm.nih.gov/22961906/

22. Braune A, Gütschow M, Engst W, Blaut M. Degradation of quercetin and luteolin by Eubacterium ramulus. Appl Environ Microbiol [Internet]. 2001 Dec [cited 2022 Nov 18];67(12):5558–67. Available from: https://pubmed.ncbi.nlm.nih.gov/11722907/

23. Cheng JR, Liu XM, Chen ZY, Zhang YS, Zhang YH. Mulberry anthocyanin biotransformation by intestinal probiotics. Food Chem [Internet]. 2016 Dec 15 [cited 2022 Nov 18];213:721–7. Available from: https://pubmed.ncbi.nlm.nih.gov/27451240/

24. Verediano TA, Stampini Duarte Martino H, Dias Paes MC, Tako E. Effects of Anthocyanin on Intestinal Health: A Systematic Review. Nutrients [Internet]. 2021 Apr 1 [cited 2022 Nov 18];13(4). Available from: https://pubmed.ncbi.nlm.nih.gov/33920564/

25. Burapan S, Kim M, Han J. Curcuminoid Demethylation as an Alternative Metabolism by Human Intestinal Microbiota. J Agric Food Chem [Internet]. 2017 Apr 26 [cited 2022 Nov 18];65(16):3305–10. Available from: https://pubmed.ncbi.nlm.nih.gov/28401758/

26. Hassaninasab A, Hashimoto Y, Tomita-Yokotani K, Kobayashi M. Discovery of the curcumin metabolic pathway involving a unique enzyme in an intestinal microorganism. Proc Natl Acad Sci U S A [Internet]. 2011 Apr 19 [cited 2022 Nov 18];108(16):6615–20. Available from: https://pubmed.ncbi.nlm.nih.gov/21467222/

27. Tan S, Rupasinghe TWT, Tull DL, Boughton B, Oliver C, McSweeny C, et al. Degradation of curcuminoids by in vitro pure culture fermentation. J Agric Food Chem [Internet]. 2014 Nov 12 [cited 2022 Nov 18];62(45):11005–15. Available from: https://pubmed.ncbi.nlm.nih.gov/25317751/

28. Gaya P, Peirotén Á, Medina M, Álvarez I, Landete JM. Bifidobacterium pseudocatenulatum INIA P815: The first bacterium able to produce urolithins A and B from ellagic acid. J Funct Foods. 2018 Jun 1;45:95–9.

29. Selma M V., Tomás-Barberán FA, Beltrán D, García-Villalba R, Espín JC. Gordonibacter urolithinfaciens sp. nov., a urolithin-producing bacterium isolated from the human gut. Int J Syst Evol Microbiol [Internet]. 2014 [cited 2022 Nov 18];64(Pt 7):2346–52. Available from: https://pubmed.ncbi.nlm.nih.gov/24744017/

30. García-Villalba R, Beltrán D, Frutos MD, Selma M V., Espín JC, Tomás-Barberán FA. Metabolism of different dietary phenolic compounds by the urolithin-producing human-gut bacteria Gordonibacter urolithinfaciens and Ellagibacter isourolithinifaciens. Food Funct [Internet]. 2020 Aug 1 [cited 2022 Nov 18];11(8):7012–22. Available from: https://pubmed.ncbi.nlm.nih.gov/32716447/

31. María M, Selma V, Beltrán D, Beltrán B, Rocío R, García-Villalba G, et al. Description of urolithin production capacity from ellagic acid of two human intestinal Gordonibacter species. Food Funct [Internet]. 2014 Jul 24 [cited 2022 Nov 18];5(8):1779–84. Available from: https://pubs.rsc.org/en/content/articlehtml/2014/fo/c4fo00092g
